# Evolutionary dynamics of the human pseudoautosomal regions

**DOI:** 10.1371/journal.pgen.1009532

**Published:** 2021-04-19

**Authors:** Bruno Monteiro, Miguel Arenas, Maria João Prata, António Amorim

**Affiliations:** 1 Institute of Investigation and Innovation in Health (i3S). University of Porto, Porto, Portugal; 2 Institute of Molecular Pathology and Immunology (IPATIMUP), University of Porto, Porto, Portugal; 3 Department of Biochemistry, Genetics and Immunology, University of Vigo, Vigo, Spain; 4 CINBIO (Biomedical Research Centre), University of Vigo, Vigo, Spain; 5 Faculty of Sciences, University of Porto, Porto, Portugal; George Washington University, UNITED STATES

## Abstract

Recombination between the X and Y human sex chromosomes is limited to the two pseudoautosomal regions (PARs) that present quite distinct evolutionary origins. Despite the crucial importance for male meiosis, genetic diversity patterns and evolutionary dynamics of these regions are poorly understood. In the present study, we analyzed and compared the genetic diversity of the PAR regions using publicly available genomic sequences encompassing both PAR1 and PAR2. Comparisons were performed through allele diversities, linkage disequilibrium status and recombination frequencies within and between X and Y chromosomes. In agreement with previous studies, we confirmed the role of PAR1 as a male-specific recombination hotspot, but also observed similar characteristic patterns of diversity in both regions although male recombination occurs at PAR2 to a much lower extent (at least one recombination event at PAR1 and in ≈1% in normal male meioses at PAR2). Furthermore, we demonstrate that both PARs harbor significantly different allele frequencies between X and Y chromosomes, which could support that recombination is not sufficient to homogenize the pseudoautosomal gene pool or is counterbalanced by other evolutionary forces. Nevertheless, the observed patterns of diversity are not entirely explainable by sexually antagonistic selection. A better understanding of such processes requires new data from intergenerational transmission studies of PARs, which would be decisive on the elucidation of PARs evolution and their role in male-driven heterosomal aneuploidies.

## Author summary

The pseudoautosomal regions (PAR1 and PAR2) of the human sex chromosomes contain the unique blocks of homologous sequences between the X and Y chromosomes that allow pairing and recombination during male meiosis. Both PARs present a distinctive transmission behavior that includes the properties of either sex-linked or autosomal regions (hence the pseudoautosomal status). However, the type and extent of evolutionary forces acting on these regions are, so far, not totally clear. In this work, we aimed to study the genetic diversity patterns in the human PARs, taking advantage of the publicly available resource of human genetic variation provided by the 1000 Genomes Project. Our results showed a higher frequency of recombination in PAR1 when compared to PAR2, as well as, a fairly uneven distribution of recombination throughout both regions. Moreover, in both PARs, low recombination segments are consistently associated with increased linkage disequilibrium and significantly different allele frequencies between the X and Y chromosomes. Despite the discrepancy of male recombination rates (obligatory at PAR1 and in ≅1% of meiosis at PAR2), both regions harbor highly enriched stretches with regard to allele frequency differences among sexes, which are not counterbalanced by recombination, raising questions about underlying evolutionary forces (e.g., selection). Since our results do not fit the expectations of a sexually antagonistic model, new data based on fast evolving markers from intergenerational transmission studies of PARs are required for the elucidation of the evolutionary behavior and crucial role of these regions in male-driven sex-chromosome aneuploidies.

## Introduction

In eutherian mammals, with only few exceptions [1,2], genetic determination of sex is based on a chromosomal heteromorphism of the XX/XY type, in which males are heterogametic [3,4]. A mutation in a gene that would become a sex-determining master is thought to be responsible for triggering an evolutionary process of stepwise loss of recombination between the ancestral autosome pair [3,5,6]. This process ultimately led to the formation of a non-recombining male specific region (MSY) [3] also involving a series of inversions in the proto-Y chromosome. To some extent recombination between the X and Y chromosomes is necessarily kept to ensure proper segregation in male meiosis [7]. This pairing is possible at a homologous sub-telomeric zone, appropriately named as pseudoautosomal region (PAR). Humans are unique among mammals in having two of these regions (PAR1 and PAR2) [8,9,10]: PAR1 encompasses 2.7 Mb in the tip of the short arm of the heterosomes, while PAR2 comprises 330 Kb at the heterosomes long arm [11]. In male meiosis, a crossover in PAR1 is mandatory for the proper disjunction of X and Y chromosomes, as indicated by the association between PAR1 deletion and total male sterility [12]. Moreover, it is believed that failing in heterosome pairing leads to aneuploid gametes, which are, however, viable and successful in producing chromosomally abnormal individuals (e.g., in Klinefelter syndrome [13]). The two PARs present very different origins and properties. PAR1 is found in most eutherian mammals and was incorporated within the last 105 million years to the pre-existing sex chromosome. In contrast, PAR2, which shows much less recombination, emerged by duplication of material from X to Y chromosome after the divergence of humans and chimpanzees, approximately 6 million years ago [4]. Indeed, PAR1 and PAR2 are not equally important to ensure euploid spermatogenesis. PAR1 seems absolutely essential as, at least, one crossover event is observed in this region during normal male meiosis. Accordingly, PAR1 male recombination rate is exceptionally elevated, ~17-fold higher than the genome-wide average [14], while the female recombination rate is only slightly over the genome average. In contrast, PAR2 apparently has a minor role since it has a much smaller recombination frequency estimated to occur at just 1% of male meiosis and extremely rarely in female gametogenesis [15]. Thus, the unevenness of meiotic recombination is sex-linked since in female meiosis the entire X chromosome recombines evenly throughout its entire length (including PARs), while on the other hand, in spermatogenesis, it occurs only at the PARs.

The heterogeneous evolutionarily past of PARs endows them with unique genetic properties. First, PAR loci display a transmission pattern that is neither purely autosomal nor sex linked. PAR alleles spend unequal amounts of time in males and females, subjecting them to sex-specific evolutionary forces [11,16]. Second, the maintenance of polymorphisms under sexually antagonistic selection (i.e., when alleles present different fitness effects in females and males) is facilitated in PARs [17,18]. Third, genetic diversity is expected to be higher in PARs because: *i*) recombination can unlink alleles affected by (purging) selection from nearby sites; *ii*) the effective population size of PARs (two copies in both males and females) is larger than in the remaining region of the X (two copies in females and one in males) or Y (just one copy in males) chromosomes [19]; *iii*) a higher recombination rate at PARs increases the local mutation rate, and consequently PARs are expected to be enriched with SNPs, CNVs and segmental duplications [14]. Actually, the existence of two PARs in humans poses an extra evolutionary paradox: since the infrequent PAR2 recombination alone does not seem sufficient to ensure balanced chromosome disjunction, it cannot be seen as a PAR1 ‘backup’, and thus other forces behind its creation and maintenance must occur. In fact, due to its low recombination rate, the entire PAR2 behaves as an (almost) heterosomal specific region, travelling most of its evolutionary time in association with either X or Y chromosome. This particular feature enables an almost independent accumulation of mutations at X and Y chromosomes associated with PAR2, leading to progressive divergence between them. Ultimately this characteristic would be reflected into asymmetries in allele frequencies and linkage disequilibrium (LD) patterns. However, comparative data on genetic diversity, differences in allele frequencies between X and Y chromosomes, LD patterns and recombination landscapes involving PAR1 and PAR2 are scarce. Hence, in this work, we present insights into the PARs complex evolutionary paths using *Homo sapiens* as a model, not only because PAR2 is exclusive of the human lineage, but also due to the availability of extensive genetic data of the population diversity of these regions. For that, we used publicly available data of human populations (the most recent release of the 1000 Genomes Project, abbreviated as 1kGP) to investigate the distribution of genetic diversity, the differences of allelic frequencies at PARs from X and Y chromosomes and to explore the pseudoautosomal regional recombination landscape, including the corresponding LD and recombination patterns.

## Results

### Distribution of genetic diversity along PARs

**Fig 1:**
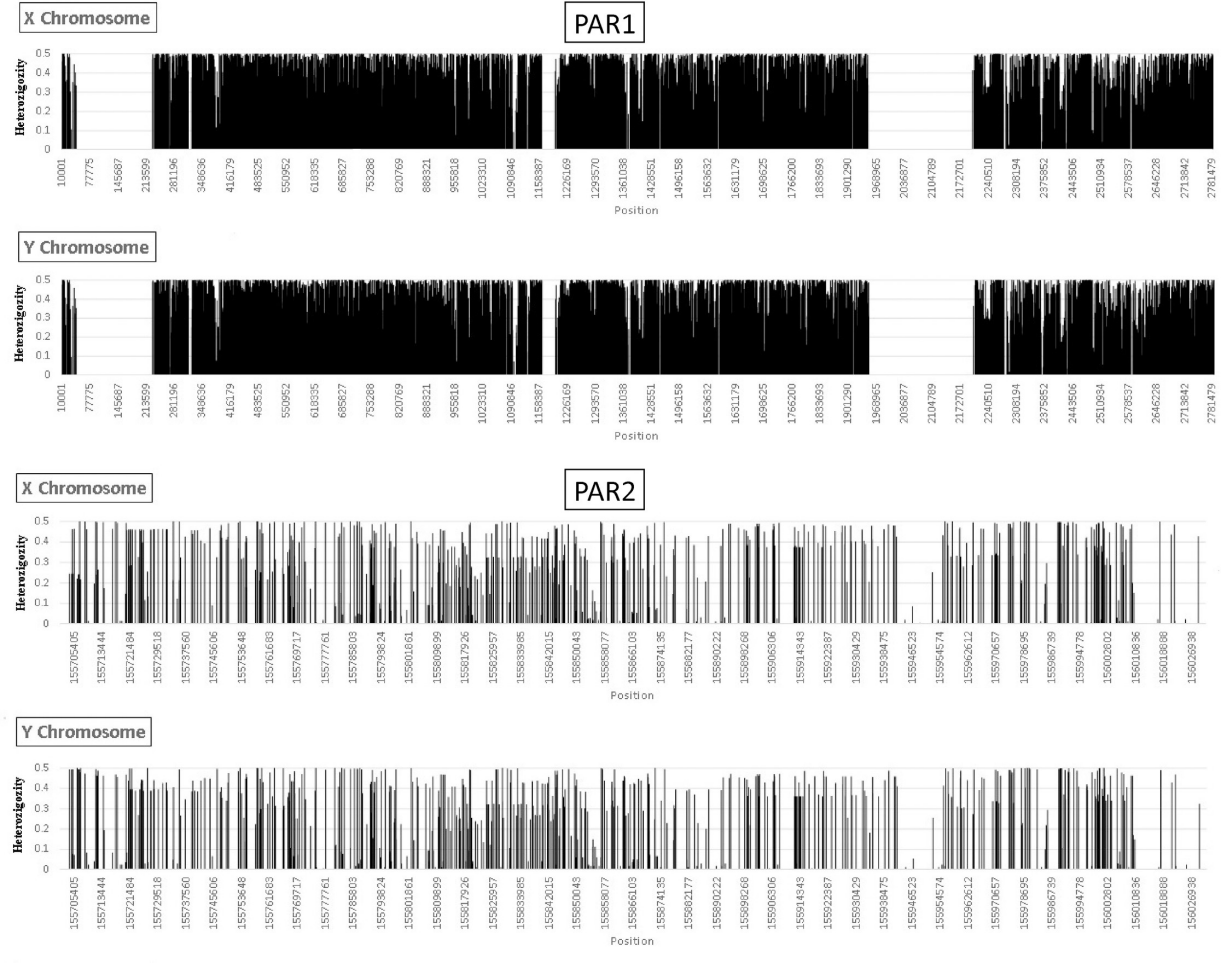
Distribution of heterozygosity along PARs at X and Y chromosomes. Data from the 1kGP African population for polymorphic and non-polymorphic positions encompassing PAR1 and PAR2 at the X and Y chromosomes.

We evaluated the patterns of genetic diversity across PAR1 and PAR2, separately for X and Y chromosomes, by calculating heterozygosity at each position (distributions shown in Fig 1). Note that since the only variation consists of SNPs, values per site (position) vary between 0 and 0.5. Indeed, we have focused on the distribution of long stretches (>10 Kbp) devoided of variable sites considering the absence of genetic diversity as a molecular signature of strong purging selection. At PAR1 we found three of those stretches: the shortest at the center of the region and the longest near the pseudoautosomal border (intervals with approximate position coordinates: 44000–228000, 1164000–1197000 and 1950000–2200000). These regions are entirely coincident, both in position and in length, between the X and Y chromosomes.

In striking contrast, at PAR2 (in both X and Y chromosomes) we did not find the latter long, monomorphic stretches. This was unexpected considering its much shorter length relative to PAR1, lower overall diversity and the need for extensive homology required for recombination. However, two well-defined long regions showing marked reduced diversity were found: one centered at an approximate position of 155949795 and the other one at the telomere.

### Allele frequency differences between sexes and between the X and Y chromosomes

**Fig 2:**
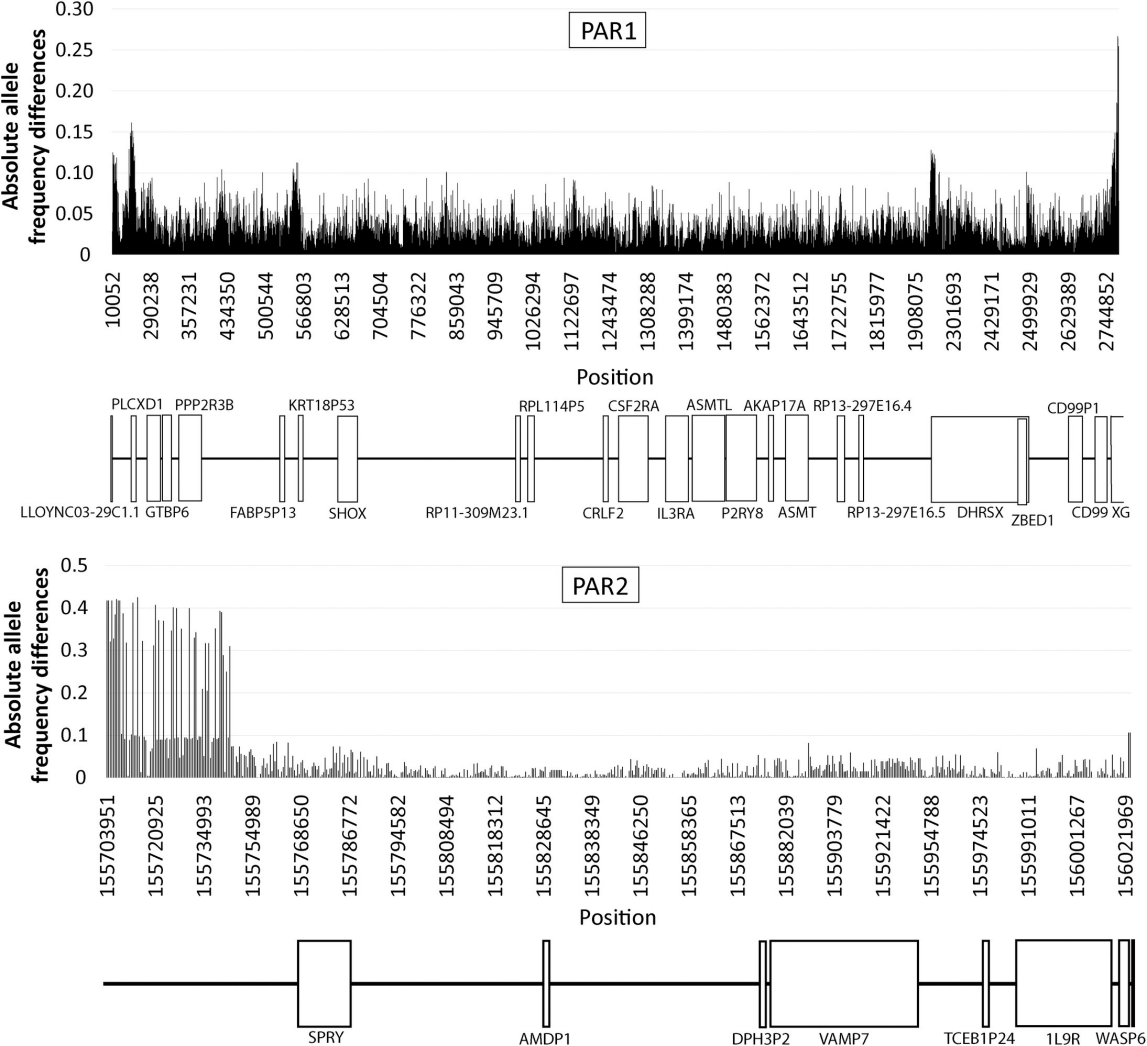
Distribution of allele frequency differences between X and Y chromosomes along PARs. Allele frequency differences between the X and Y chromosomes for SNPs at PAR1 and PAR2 from the 1kGP African population.

**Fig 3:**
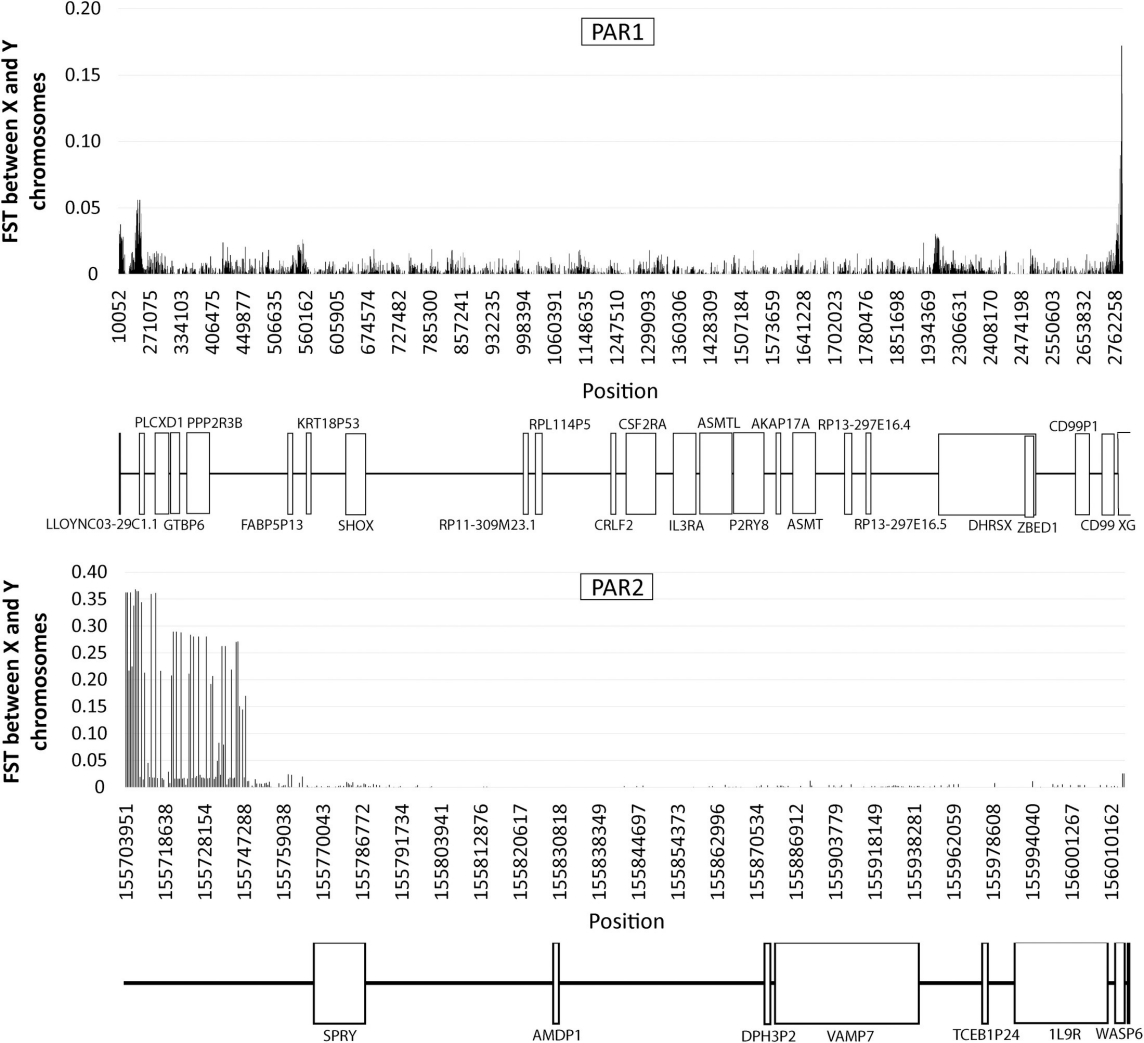
Distribution of Fst between X and Y chromosomes along PARs. Fst estimates between the X and Y chromosomes for SNPs at PAR1 and PAR2 from the 1kGP African population.

Allelic frequency differences at PARs between the X and Y chromosomes for the 1kGP African population data are shown in Fig 2. The same comparison but performed between females and males, is displayed in S1 and S2 Figs. On average, allele frequency differences are small (rarely above 0.1) and are concentrated in blocks, a distribution found in both genic and intergenic regions. This analysis was also performed with the 1kGP African population using Fst as a measure of the X/Y substructure of PARs gene pools [20] (Fig 3). A particular feature in PAR1 deserves a special note, as it involves a very peculiar gene (XG) that is partially sex-linked and partially pseudoautosomal (the first 3 exons of XG are inside PAR1 while the remaining 7 are located in the X-specific region [21]). At the zone of XG, nearest to PAR1 boundary, we observed the highest Fst values and allele frequency differences between the X and Y, which might be indicative of especially low recombination rate in this region.

At PAR2, the patterns of Fst and allele frequency differences between chromosomes and sexes are similar to those in PAR1, namely: *i*) the magnitude of allele frequency differences in PAR2 is considerably lower than in the flanking non-PAR; *ii*) similar distribution of Fst and allele frequency differences in genic and intergenic regions; and *iii*) accumulation of SNPs with highest Fst and chromosome (and sex) divergent allele frequencies at the vicinity of PAR2 boundary. Also, in Africans, the distribution of Fst and the pattern of allele frequency differences between sexes in PAR1 and PAR2 were quite similar.

Furthermore, we evaluated if the patterns that emerged from the observed absolute allele frequency differences between males and females (S3 Fig) persist when the analysis is limited to the statistically significant differences (according to Fisher’s test). The results of that reanalysis showed patterns that concurred entirely with those obtained in the extended set (S4 Fig).

In addition, when confirmatory tests for Hardy-Weinberg equilibrium (HWE) departures were performed separately for both males and females, a good correlation was detected between χ^2^ values and differences in allele frequencies, particularly in males (S5 Fig).

### Evaluation of linkage disequilibrium

The distribution of LD along PARs in X and Y chromosomes of the African 1kGP population set is presented in S6 and S7 Figs and is also expanded for regions near the pseudoautosomal border (S8 Fig). The equivalent results according to the sex are shown in S9–S11 Figs and in S12–S14 Figs.

PAR1 revealed quite identical LD patterns in X and Y chromosomes as well as in both sexes. The whole region is occupied by relatively small blocks of high (*r*^2^ > 0.75) or moderate (0.4 < *r*^2^ < 0.75) LD separated by intervals with low association. The longest block with high LD encompasses around 10 kb inside the DHRSX gene, which is larger than the longest block described in the literature for PAR1 (inside the SHOX gene [22]). The second longest LD block that we identified, spans ~7 kb inside the ASMTL gene. Heatmaps showing the distribution of LD blocks within PAR1 (S6 and S9–S11 Figs) revealed that the length of strong LD blocks tends to increase from the region next to the telomere towards the sex-specific region, in both sexes. Surprisingly, the above mentioned zone comprising part of the XG gene (S1 Table), which includes many SNPs presenting high Fst and statistically significant allele frequency differences between X and Y, does not show the expectedly high LDs; a feature that deserves a dedicated study.

Similarly to PAR1, PAR2 also showed a block-like pattern of LD considering either sexes or chromosomes (S7 and S12–S14 Figs), but showed *r*^*2*^ values generally higher than in PAR1, particularly for the block at the pseudoautosomal border in males (S8 Fig). Concerning the LD block near the telomere, it encompasses 4 out of the 7 genes located in PAR2 (VAMP7, TCEB1B24, IL9R and WASP6). In addition, the highest level of LD observed within the VAMP7 gene (average *r*^*2*^ = 0.4 with 20% of values above 0.75) is especially remarkable since it is one of the genes located in the telomeric *Theria* X before the Y inversions occurred [8].

### Evaluation of the recombination rate

**Fig 4:**
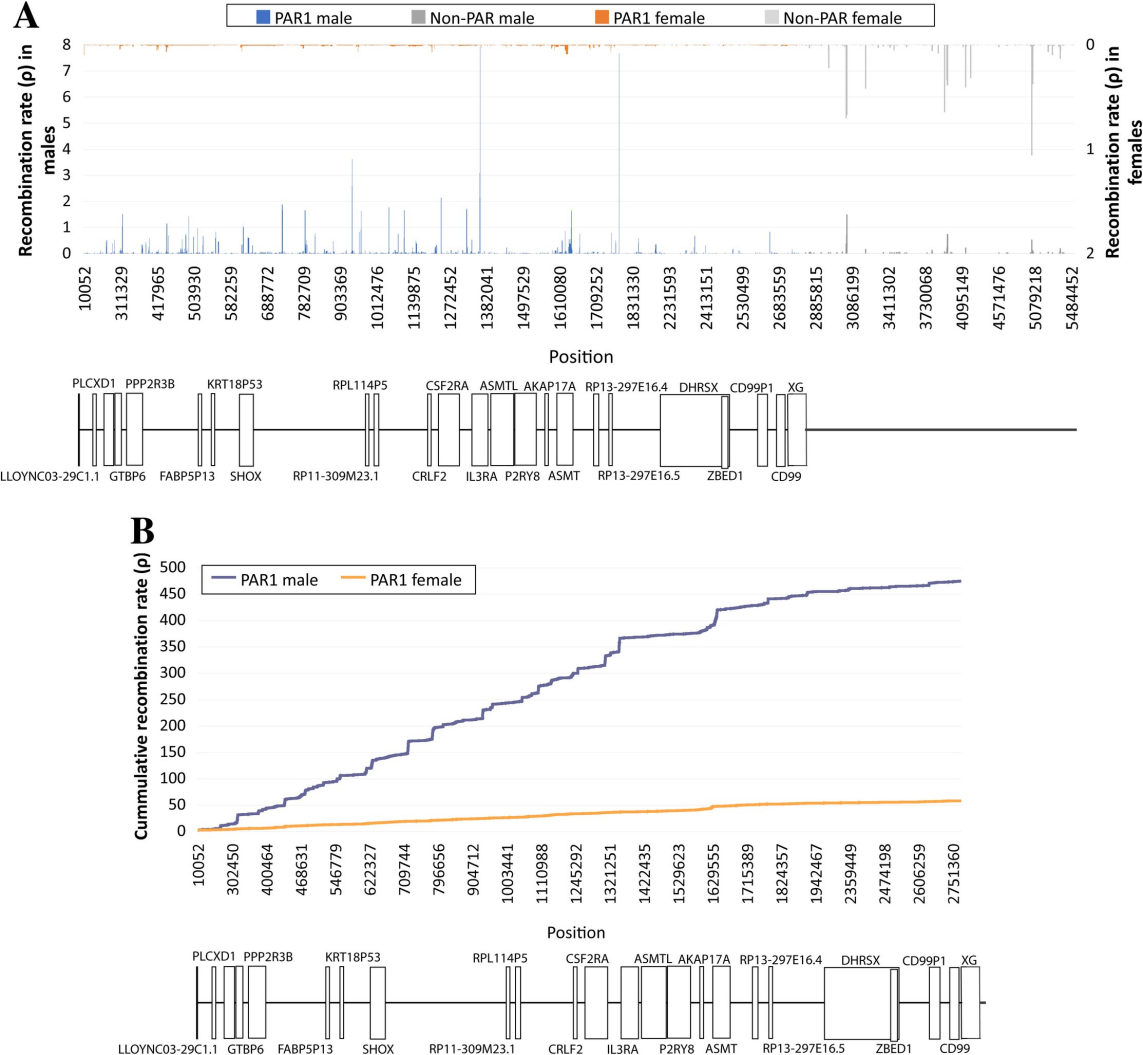
Estimated recombination rate in PAR1. Average (*A*) and cumulative (*B*) recombination rate (ρ) at PAR1 and the immediately adjacent neighboring sex-specific region based on 180 female (orange) and male (blue) individuals from the 1kGP African population.

We estimated the population recombination rate (ρ) across the two PARs in males and females. Concerning PAR1 (Fig 4A), males systematically showed high values of ρ, suggesting intense recombination along the entire region. Still, recombination was rather unevenly distributed since several recombination hotspots were found separated by regions with lack or reduced recombination. In the flanking sex-specific region some recombination is still observed which decreases with the reduction of distance from PAR1; a finding that can be expected since the PAR1 boundary does not show an abrupt decrease in X/Y homology with the Y chromosome [23]. In females, recombination in PAR1 is much lower (almost negligible) in comparison to males (very small estimates might just represent background noise intrinsic to the recombination analysis [24,25]). This difference in PAR1 recombination landscape between males and females is in accordance with the mandatory recombination at PAR1 for normal chromosomal segregation in males, whereas in females, the two X-chromosomes can pair and exchange DNA along their entire extension [14,15]. PAR1 cumulative recombination rate in males is presented in Fig 4B. Results show an interspersed pattern of hot and cold spots with recombination especially intense from the telomeric extremity to the center of the region and a progressively reduce towards the pseudoautosomal border.

As expected, recombination and LD patterns are similar. For instance, the LD blocks located at intervals 569419–613324, 1347353–1658065, 2467952–2479037 and 2215259–2385255 bp correspond precisely to regions characterized by low recombination. This is also in line with the map of allele frequency differences between sexes and chromosomes, which are higher in PAR1 segments presenting low recombination and high LD (such as the complex *locus* DHRSX-ZBED1, one of the richest *loci* in SNPs with sex specific frequencies, except for the short region where the two genes overlap). Consistently, this short region escapes the LD block that dominates the remaining DHRSX-ZBED1 *locus*. Another example can be found at the beginning of the XG gene that presents high allele frequency differences and low recombination rate.

Concerning the identified recombination hotspots, although most of them reside in intergenic regions, some are located inside genes (the higher recombination rates were found at IL3RA, ASMT and GBTP6). Recombination was much lower in other genes, such as SHOX (previously considered as a recombination hotspot [26]), PLCXD1 and in particular PPP2R3R, DHRSX and CD99P1. Accordingly, the three latter genes showed strong LD blocks (S6 Fig).

**Fig 5:**
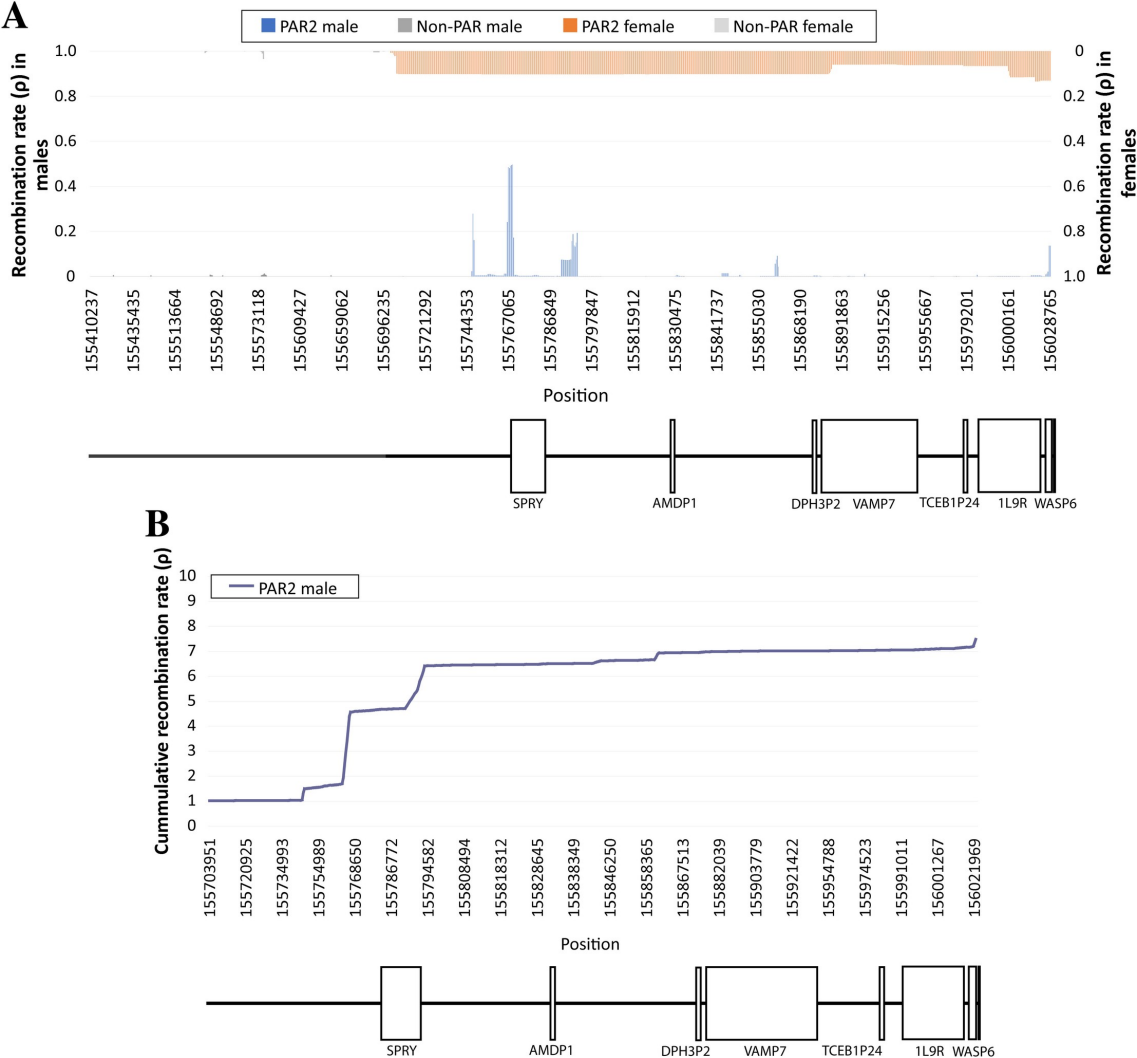
Estimated recombination rate in PAR2. Average (*A*) and cumulative (*B*) recombination rate (ρ) at PAR2 and the immediately adjacent neighboring sex-specific region based on 180 female (orange) and male (blue) individuals of the 1kGP African population.

Recombination in PAR2 is disproportionately infrequent in comparison with PAR1, especially in females (Fig 5A), where ρ was below 0.1 along the entire region and indistinguishable from the background noise of the estimation method. In males, although several peaks of recombination rate are observed, all of them are located at intergenic regions. The highest one, immediately upstream from the SPRY3 gene, corresponds to a ∼1 kb stretch previously identified as a recombination hotspot [27]. Our estimated recombination map essentially agrees with the one presented by Sarbajna *et al*. [27], but in addition it covers the proximal and distal tips of PAR2 that were not addressed. In the distal extremity we identified a peak of recombination activity, whereas near the pseudoautosomal border (which concentrates the most extreme allele frequency differences between sexes and chromosomes) we found lack of recombination.

The cumulative recombination graph in PAR2 (Fig 5B) displays values of ρ > 0.2 and for males only, since recombination is too low in females to be represented) shows a concentration of recombination activity at the proximal half, which matches the first series of LD blocks separated by regions without LD (male LD heatmap in S12 Fig). Conversely, in the stretch with more intense male recombination (from 155,744,353 bp to 155,794,729 bp) LD is weak.

Despite the apparent absence of recombination in the female PAR2, the LD distribution is similarly structured in both sexes. Most likely this means that the X chromosome that they obligatorily received from males, in each generation, probably causes the LD pattern observed in females where recombination frequency is already low.

Our results confirm the occurrence of recombination in PAR2, mainly in males, and at a much lower rate than in PAR1, which is in agreement with previous reports pointing out that only around 1% of recombination occurs in male meiosis [14,15]. As a control check, we performed an analysis to evaluate the accuracy of our genetic map quantified with ρ [28] in comparison with genetic linkage estimated in centiMorgan (cM) lengths reported in the literature. The published ratios between male and female PAR1 cM lengths varied from 9 to 15 and between male PAR1 and male PAR2 from 54 to 79 (see S2 Table and references therein). We estimated ratios of 9 and 73, respectively, falling comfortably within the ranges inferred using linkage/pedigree analyses. This agreement supports our landscape of recombination rate inferred for PAR1 and PAR2, which in addition relies on a higher number of markers (12,904 and 634 SNPs for PAR1 and PAR2, respectively; S3 Table).

## Discussion

The two PAR segments of the human sex chromosomes share the unique property of pairing and exchanging genetic information during male meiosis. However, PAR1 and PAR2 differ in many aspects, namely in size (PAR1 is approximately eight times longer than PAR2), evolutionary history (in contrast to PAR1, PAR2 was X-transposed to the Y chromosome after the human/chimpanzee divergence) and, more importantly, in the successful role of X-Y chromosomes pairing (very well documented for PAR1, but still uncertain for PAR2), which, in turn, is critically associated with chromosomal aneuploidy and male fertility.

Recombination (and its suppression) is recognized as a relevant process in the evolution of sex chromosomes in mammals. Likewise, we postulated that meiotic recombination could be a main driver in patterns of genetic diversity in human PAR1 and PAR2 (taking into account the major difference between rates of occurrence at these regions) in male and female gametogenesis. Under this hypothesis, we revisited the recombination pattern landscape in both regions and some of their features, namely, genetic diversity, prioritizing the poorly explored till date (i.e., distribution of gene diversity, sex and chromosome allele frequency divergences and HWE) or required to be further elucidated through high-resolution approaches (i.e., LD distribution patterns). We found that in both PARs, human meiotic recombination follows the well-established principle of a non-randomly distributed process. Instead, it is intensely punctuated with hotspot clusters surrounded by large DNA stretches where recombination is suppressed [29,30,31,32]. Here, we present for PAR1 and PAR2 a fine-scale distribution of recombination hotspots that meets and extends previously published data [15,33,34,35,36,37,38,39]. We obtained a good agreement between the hotspots inferred from past recombination and those previously published either based on population [35] or pedigree analyses [15,36,37,38,39]. The newly detected potential hotspots, in particular in PAR1, raise new challenges to be further investigated through experimental strategies (i.e., sperm-based crossover analyses [40]). In agreement with previous studies [41], we confirmed the role of PAR1 as a crucial region of male-specific recombination, but also that male recombination occurs in PAR2 at an insufficient (low) rate [42] to permeate patterns of diversity in the region between both sex chromosomes. Hence, to our knowledge, we show for the first-time evidence that low recombination seems, nevertheless, to affect the maintenance of sex and chromosomal specific allele frequencies in both PARs.

We further hypothesized that the low recombination rate at PARs, in female meiosis, could enable the independent accumulation of mutations at the regions in both X and Y chromosomes. This would lead to progressive divergence among chromosomes in the two sexes, in particular, where recombination is infrequent (specifically in PAR2). We then predicted that this divergence would be reflected in genetic diversity, allele frequencies and linkage disequilibrium differential patterns between heterosomes. Based on diverse analytical approaches (i.e., distribution of recombination rates, patterns of heterozygosity, allele frequency differences between sexes and chromosomes, and distributions of Fst) our results confirmed these expectations. Indeed, we demonstrated that both PARs harbor zones significantly and coincidently heterogeneous concerning the cited genetic statistics and that divergent allele frequencies are abundantly present.

Since both human PARs show significant differences in allele frequencies between sexes and chromosomes and recombination seems an insufficient homogenizing process, it remains to be clarified which evolutionary forces may favor the observed asymmetry. We strongly believe that sexually antagonistic selection and transmission bias are likely to be involved [18,43]. Nevertheless, our results seem to disprove the theory that rare recombination events are enough to prevent genetic differentiation among sex chromosomes [44,45] and, as well, do not support the hypothesis that most PAR genes evolve similarly to autosomal rather than to sex-linked genes (unless recombination is very rare) [46].

Also in contradiction to our results are the predictions that divergence between the X and Y chromosomes occurs only if the strength of sexually antagonistic selection is higher than the recombination rate (with *r* = 1% a sex-specific selection coefficient of *s* = 0.03 is needed to generate an Fst of ∼0.1) and thus that the region of X–Y divergence should be confined to a small area near the pseudoautosomal border, unless male *r* is low [47]. Therefore, our results seem to support the idea that the role of genes under sexually antagonistic selection in sex chromosome evolution might have been overemphasized [48]. In addition, the heterozygosity distribution that we found at both PARs does not meet the expectation that polymorphisms should be more prevalent at loci in the region closer to the pseudoautosomal border [4]. Indeed, the coincidence of homozygosity with recombination hotspots suggests a likely presence of strong purifying selection maintaining the homology required for pairing. More generally, we also showed (considering PAR2) that high recombination rates do not seem to be essential to ensure some stability of the process over evolutionary time [4].

Clearly, many conflicting issues remain unsolved, but we strongly agree with the statement *«This is a worthy area for future theoretical development»* [4] because more and highly informative data is necessary. Further research using fast evolving markers (i.e., microsatellites) and studies of intergenerational transmission [49,50] would be convenient to enlighten these questions. Another investigation that we deem important is a detailed study focused on the (few) genes located at PARs, including the comparative analyses of coding, non-coding and regulatory regions.

## Materials and methods

### Description of the study data

We collected genetic data of X and Y chromosomes from phase 3 of the 1000 Genomes Project (1kGP) [51]. The data consisted of full, phased nucleotide sequences (extracted in variant call format, *vcf*) belonging to 2,504 individuals from 26 populations (S4 Table) and aligned the respective sequences to the CRCh38 reference genome.

A quality control filter was applied using *VCFtools* [52]. From the initial dataset, none of the individuals were excluded since no related individuals were found and the maximum missing genotype rates per individual were low (0.94% for the Y chromosome and 0% for the X chromosome). Duplicated or triplicated information per *locus*, insertions and deletions (InDels), copy number variants (CNVs) and variant positions with more than 2 alleles per *site* were excluded. Only SNPs with a conservative value of Minimum Allele Frequency (MAF) above 0.05 were considered, to minimize phasing, imputation and genotyping errors critical for rare alleles [53], with exception for genetic diversity distribution calculations. The number of markers used in the analyses was 12,904 for PAR1 and 634 for PAR2 (S3 Table).

### Estimation of allelic frequencies

Allele frequencies were estimated by counting with *VCFtools*. Estimates were obtained for the two heterosomes and sexes in the different continental population groups (S5 Table). We presented the absolute differences of allele frequencies between heterosomes per site (considering the annotated position) in PAR1 and PAR2.

### Estimation of heterozygosity

The estimation of heterozygosity per *locus*, for both PARs of the two heterosomes, in the African population was performed with *Arlequin v3*.*5*.*2*.*2* [54].

### Detection of allele frequency differences between sexes and chromosomes, and departures from Hardy-Weinberg Equilibrium expectations

In order to evaluate if allele frequencies in males and females significantly differ, we performed a Fisher exact test assuming as significant a *p* value below 0.05. We presented the statistically significant differences of allele frequencies between the two sexes using a heatmap built with *R* [55].

Deviations from *Hardy-Weinberg Equilibrium* (HWE) expectations were evaluated through *χ*^*2*^ tests implemented in *VCFtools* and both sexes were independently assessed. Results are presented for *χ*^*2*^ values above 3.841 (*p* < 0.05 for 1 degree of freedom).

### Estimates of genetic differentiation (Fst)

Genetic differentiation based on Fst was estimated using *Genepop v4*.*7* [56] between the two heterosomes for both PAR1 and PAR2 in the African population data set.

### Analysis of linkage disequilibrium

Linkage disequilibrium (LD) was evaluated through *r*^*2*^ (that evaluates the non-random association of alleles at different loci in a given population) implemented in the *VCFtools* package. We presented the *r*^*2*^ estimates obtained for heterosomes, for males and females, using a heatmap built with the *BMapBuilder3B* framework [57]. LD was assessed between every SNP in the PAR2 region. For PAR1, the window size considered for LD calculations was set to the maximum SNP distance observed in PAR2 (324,815 bp).

### Recombination analysis

In order to perform the recombination analysis, we converted the format of the data from *vcf* to *fasta* using the *VCFlib* package [58]. We estimated the population recombination rate per site ρ *= 4Nr* (where *N* is the effective population size and *r* is the recombination rate per site) using the well-established framework *LDhat* [59]. This framework is computationally limited to an analysis of a maximum of 180 sequences (due to the calculation of the lookup table with the coalescent likelihood of all possible two-locus haplotype configurations [59,60]) and thus we randomly selected a sample of 180 African sequences. We calculated the average and cumulative ρ among sites. The analysis was independently performed for each heterosome and in both sexes.

## Supporting information

S1 Table Genes located in the studied genomic regions. The table shows the genomic (position) coordinates (in base pairs) of the genes located in PAR1 and PAR2.
(PDF)

S2 Table Recombination estimates comparisons between the present study and the selected literature. Recombination rate estimates published in other studies (including additional information) compared with the estimates obtained in the present study.
(PDF)

S3 Table Genetic markers. Number of markers from different chromosomal regions used for the analysis before and after quality control filtering.
(PDF)

S4 Table Studied populations. List of the 1kGP populations analyzed in this study.
(PDF)

S5 Table Individuals analyzed per population. Number and sex of individuals from each population analyzed in this study.
(PDF)

S1 Fig Distribution of allele frequency differences between sexes for PAR1. Absolute allele frequency differences between males and females for SNPs in PAR1 (black) and its flanking portion of the sex-specific region (grey) in the 1kGP African population data.
(PDF)

S2 Fig Distribution of allele frequency differences between sexes for PAR2. Absolute allele frequency differences between males and females for SNPs in PAR2 (black) and its flanking portion of the sex-specific region of the chromosome (grey) in the 1kGP African population data.
(PDF)

S3 Fig Distribution of allele frequency differences between sexes for PARs in worldwide populations. Absolute allele frequency differences between males and females for SNPs in the PARs (blue) and flanking portions (orange) in the five 1kGP super-populations.
(PDF)

S4 Fig Significant allele frequency differences between sexes for PAR1 and PAR2. Heatmaps generated from allele frequency statistically significant differences between sexes for PAR1 (A) and PAR2 (B) in the different 1kGP super-populations. AMR: Admixed Americans; EAS: East Asians; SAS: South Asians; EUR: Europeans; AFR: Africans. The blue tones correspond to the range of *p* values of the Fisher test obtained for allele frequencies differences, according to the color key in the figure.
(PDF)

S5 Fig Distribution of HWE departures for PAR1 and PAR2. *χ*^2^ values of HWE tests for PAR1 (A) and PAR2 (B) associated to *p* < 0.05 in males (bottom in the plots) and females (top in the plots). Statistically significant departures in both sexes are represented in blue and orange bars for males and females, respectively. Statistically significant departures in one sex only are shown in black bars.
(PDF)

S6 Fig Linkage disequilibrium heatmap*s* for PAR1 at the X and Y chromosome. LD heatmaps based on *r*^*2*^ values for PAR1 at the X (*A*) and Y (*B*) chromosomes from the 1kGP African population. Each tone corresponds to different ranges of *r*^*2*^ values as represented in the color legend of the figure.
(PDF)

S7 Fig Linkage disequilibrium heatmap*s* for PAR2 at the X and Y chromosomes. LD heatmaps based on r^2^ values for PAR2 at the X (*A*) and Y (*B*) chromosomes from the 1kGP African population. Each tone corresponds to different ranges of *r*^*2*^ values as represented in the color legend of the figure.
(PDF)

S8 Fig Linkage disequilibrium heatmaps for segments of the PARs nearest to the pseudoautosomal borders at the X and Y chromosomes. LD heatmaps based on *r*^*2*^ values at the X and Y chromosomes from the 1kGP African population. Each tone corresponds to different ranges of *r*^*2*^ values as represented in the color legend of the figure.
(PDF)

S9 Fig Linkage disequilibrium heatmap*s* for PAR1 in females and males. LD heatmaps based on *r*^*2*^ values in PAR1 at females (*A*) and males (*B*) from the 1kGP African population. Each tone corresponds to different ranges of *r*^*2*^ values as represented in the color legend of the figure.
(PDF)

S10 Fig Linkage disequilibrium heatmap for PAR1 and the pseudoautosomal border region in females. LD heatmap based on r^2^ values in PAR1 and the pseudoautosomal border region in females from the 1kGP African population. Each tone corresponds to different ranges of *r*^*2*^ values as represented in the color legend of the figure.
(PDF)

S11 Fig Linkage disequilibrium heatmap for PAR1 and the pseudoautosomal border region in males. LD heatmap based on r^2^ values for PAR1 and the pseudoautosomal border region in males from the 1kGP African population. Each tone corresponds to different ranges of *r*^*2*^ values as represented in the color legend of the figure.
(PDF)

S12 Fig Linkage disequilibrium heatmap*s* for PAR2 in females and males. LD heatmaps based on r^2^ values for PAR2 at female (*A*) and male (*B*) chromosomes from the 1kGP African population. Each tone corresponds to different ranges of *r*^*2*^ values as represented in the color legend of the figure.
(PDF)

S13 Fig Linkage disequilibrium heatmap for PAR2 and the pseudoautosomal border region in females. LD heatmap based on *r*^*2*^ values for PAR2 and the pseudoautosomal border region in females from the 1kGP African population. Each tone corresponds to different ranges of *r*^*2*^ values as represented in the color legend of the figure.
(PDF)

S14 Fig Linkage disequilibrium heatmap for PAR2 and the pseudoautosomal border region in males. LD heatmap based on *r*^*2*^ values for PAR2 and the pseudoautosomal border region in males from the 1kGP African population. Each tone corresponds to different ranges of *r*^*2*^ values as represented in the color legend of the figure.
(PDF)
