# Supplementary material for: Evolutionary dynamics of the human pseudoautosomal regions
Source: PLoS Genet. 2021 Apr 19;17(4):e1009532. doi: 10.1371/journal.pgen.1009532 (PMC8084340; doi:10.1371/journal.pgen.1009532)
Supplement: S1 Table — The table shows the genomic (position) coordinates (in base pairs) of the genes located in PAR1 and PAR2. (PDF) [file pgen.1009532.s001.pdf]

PAR1

Gene/pseudogene	Initial bp position	Final bp position
LL0YNC03-29C1.1	253743	255091
PLCXD1	276322	303356
GTPBP6	304529	318787
LINC00685	320990	321851
PPP2R3B	333963	386955
FABP5P13	523775	524102
KRT18P53	545236	545352
SHOX	624344	659411
RP11-309M23.1	990221	994365
RPL14P5	1008503	1010101
CRLF2	1187549	1212723
CSF2RA	1268800	1310381
IL3RA	1336616	1382689
ASMTL	1403139	1453762
P2RY8	1462572	1537107
AKAP17A	1591593	1602514
ASMT	1615001	1643081
RP13-297E16.4	1732584	1755985
RP13-297E16.5	1767347	1768776
DHRSX	2219516	2502805
ZBED1	2486414	2500967
CD99P1	2609348	2657229
CD99	2691179	2741309
XG	2752050	2815927

PAR2

Gene/pseudogene	Initial bp position	Final bp position
SPRY3	155767812	155782459
AMDP1	155828585	155829576
DPH3P2	155875636	155875885
VAMP7	155881293	155943769
TCEB1P24	155978992	155979325
IL9R	155997581	156010817
WASH6P	156020826	156025710
