# Supplementary material for: Evolutionary dynamics of the human pseudoautosomal regions
Source: PLoS Genet. 2021 Apr 19;17(4):e1009532. doi: 10.1371/journal.pgen.1009532 (PMC8084340; doi:10.1371/journal.pgen.1009532)
Supplement: S2 Table — Recombination rate estimates published in other studies (including additional information) compared with the estimates obtained in the present study. (PDF) [file pgen.1009532.s002.pdf]

PAR1			PAR2			Samples	Reference	Ratio: male(PAR1)/ female(PAR1)	Ratio: male(PAR1)/ male(PAR2)
Nº of markers	Recombination*		Nº of markers	Recombination*					
	Male	Female		Male	Female				
3	50	4	-	-	-	8 CEPH families	[1]	13	-
5	49.9	4.18	-	-	-	44 CEPH families	[2]	12	-
11	49	4	-	-	-	38 CEPH families	[3]	12	-
6	11.7	.8	-	-	-	40 CEPH and 146 deCODE families	[4]	15	-
1400	38	3.8	140	0.7	0	269 unrelated individuals	[5]	10	54
23	55	6	6	1	0	28 CEPH pedigrees	[6]	9	55
9	55.3	-	1	0.7	-	4 sperm donors	[7]	-	79
12904	472.1**	55.2**	634	6.5**	0**	180 unrelated individuals	Present study**	9	73

*Recombination in cM units, except for our results** that are present in ρ cumulative.

**Results from our analysis

Note: Data from references 1,2,3,4,5 and 7 were retrieved from the table 1 of: “Flaquer A, Rappold GA, Wienker TF, Fischer C. The human pseudoautosomal regions: a review for genetic epidemiologists. Eur J Hum Genet. 2008;16: 771-779”.

References:

1. Rouyer F, Simmler MC, Johnsson C, Vergnaud G, Cooke HJ, Weissenbach J. A gradient of sex linkage in the pseudoautosomal region of the human sex chromosomes. Nature. 1986;319: 291–296.
2. Page DC, Bieker K, Brown LG, Hinton S, Leppert M, Lalouel JM, et al. Linkage, physical mapping, and DNA sequence analysis of pseudoautosomal loci on the human X and Y chromosomes. Genomics. 1987;1: 243–256.
3. Henke A, Fischer C, Rappold GA. Genetic map of the human pseudoautosomal region reveals a high rate of recombination in female meiosis at the Xp telomere. Genomics. 1993;18: 478–485.
4. Kong X, Murphy K, Raj T, He C, White PS, Matise TC. A combined linkage – physical map of the human genome. Am J Hum Genet. 2004;75: 114–1148.
5. Consortium IH: A haplotype map of the human genome. Nature. 2005;437: 1299–1320.
6. Flaquer A, Fischer C, Wienker TF. A new sex-specific genetic map of the human pseudoautosomal regions (PAR1 and PAR2). Hum Hered. 2009;68(3): 192-200.
7. Lien S, Szyda J, Schechinger B, Rappold G, Arnheim N. Evidence for heterogeneity in recombination in the human pseudoautosomal region: high resolution analysis by sperm typing and radiation-hybrid mapping. Am J Hum Genet. 2000;66: 557–566.
