# Supplementary material for: Evolutionary dynamics of the human pseudoautosomal regions
Source: PLoS Genet. 2021 Apr 19;17(4):e1009532. doi: 10.1371/journal.pgen.1009532 (PMC8084340; doi:10.1371/journal.pgen.1009532)
Supplement: S3 Table — Number of markers from different chromosomal regions used for the analysis before and after quality control filtering. (PDF) [file pgen.1009532.s003.pdf]

	Initial number of variants/markers in 1000 genomes project	Final number of markers after quality control	Final number of markers per base-pair
**PAR1**	100714	12904	1 SNP per 215 bp
**PAR2**	9849	634	1 SNP per 520 bp
**X-specific**	3357530	224640	1 SNP per 681 bp
**Y-specific**	62042	1483	1 SNP per 36484 bp
