# Supplementary material for: Evolutionary dynamics of the human pseudoautosomal regions
Source: PLoS Genet. 2021 Apr 19;17(4):e1009532. doi: 10.1371/journal.pgen.1009532 (PMC8084340; doi:10.1371/journal.pgen.1009532)
Supplement: S4 Table — List of the 1kGP populations analyzed in this study. (PDF) [file pgen.1009532.s004.pdf]

**AFR – Africans**	YRI – Yoruba in Ibadan, Nigeria<br>LWK – Luhya in Webuye, Kenya<br>GWD – Gambian in Western Divisions in the Gambia<br>MSL – Mende in Sierra Leone<br>ESN – Esan in Nigeria<br>ASW – Americans of African Ancestry in SW USA<br>ACB – African Caribbeans in Barbados
**EUR– Europeans**	CEU – Utah Residents (CEPH) with Northern and Western European Ancestry<br>TSI – Toscani in Italia<br>FIN – Finnish in Finland<br>GBR – British in England and Scotland<br>IBS – Iberian Population in Spain
**SAS – South Asians**	GIH – Gujarati Indian from Houston, Texas<br>PJL – Punjabi from Lahore, Pakistan<br>BEB – Bengali from Bangladesh<br>STU – Sri Lankan Tamil from the UK<br>ITU – Indian Telugu from the UK
**EAS – East Asians**	CHB – Han Chinese in Beijing, China<br>JPT – Japanese in Tokyo, Japan<br>CHS – Southern Han Chinese<br>CDX – Chinese Dai in Xishuangbanna, China<br>KHV – Kinh in Ho Chi Minh City, Vietnam
**AMR – Admixed American**	MXL – Mexican Ancestry from Los Angeles USA<br>PUR – Puerto Ricans from Puerto Rico<br>CLM – Colombians from Medellin, Colombia<br>PEL – Peruvians from Lima, Peru
