# Supplementary material for: Evolutionary dynamics of the human pseudoautosomal regions
Source: PLoS Genet. 2021 Apr 19;17(4):e1009532. doi: 10.1371/journal.pgen.1009532 (PMC8084340; doi:10.1371/journal.pgen.1009532)
Supplement: S5 Table — Number and sex of individuals from each population analyzed in this study. (PDF) [file pgen.1009532.s005.pdf]

	AFR	EUR	EAS	SAS	AMR
X-female	342	263	260	229	177
X-male	319	240	244	260	170
Y-male*	319	240	244	260	170

*Information of the Y chromosome for the male individuals.
