# Supplementary figures and images for: Evolutionary dynamics of the human pseudoautosomal regions
Source: PLoS Genet. 2021 Apr 19;17(4):e1009532. doi: 10.1371/journal.pgen.1009532 (PMC8084340; doi:10.1371/journal.pgen.1009532)

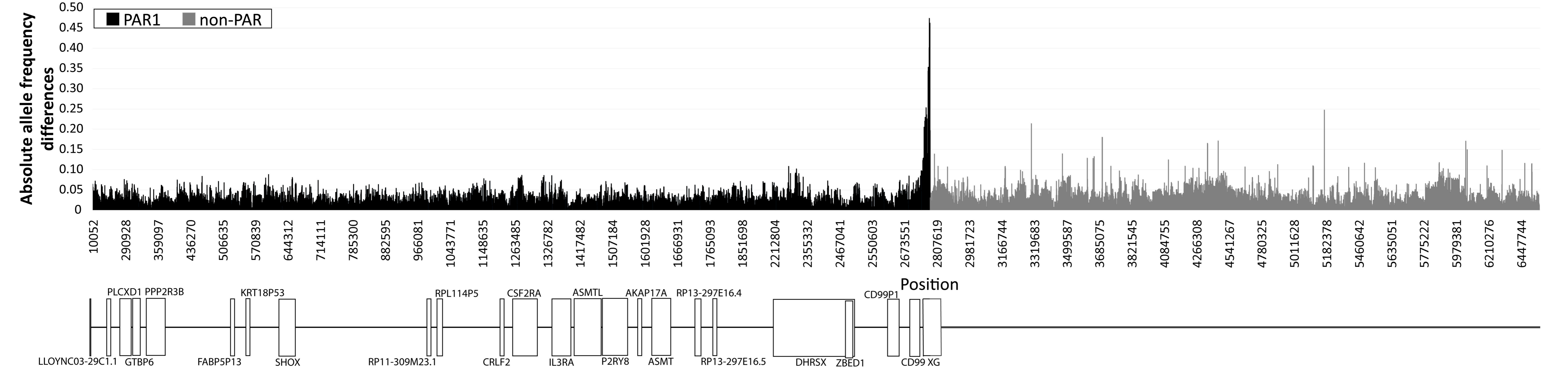

Supplement: S1 Fig — Absolute allele frequency differences between males and females for SNPs in PAR1 (black) and its flanking portion of the sex-specific region (grey) in the 1kGP African population data. (PDF) [file pgen.1009532.s006.pdf]

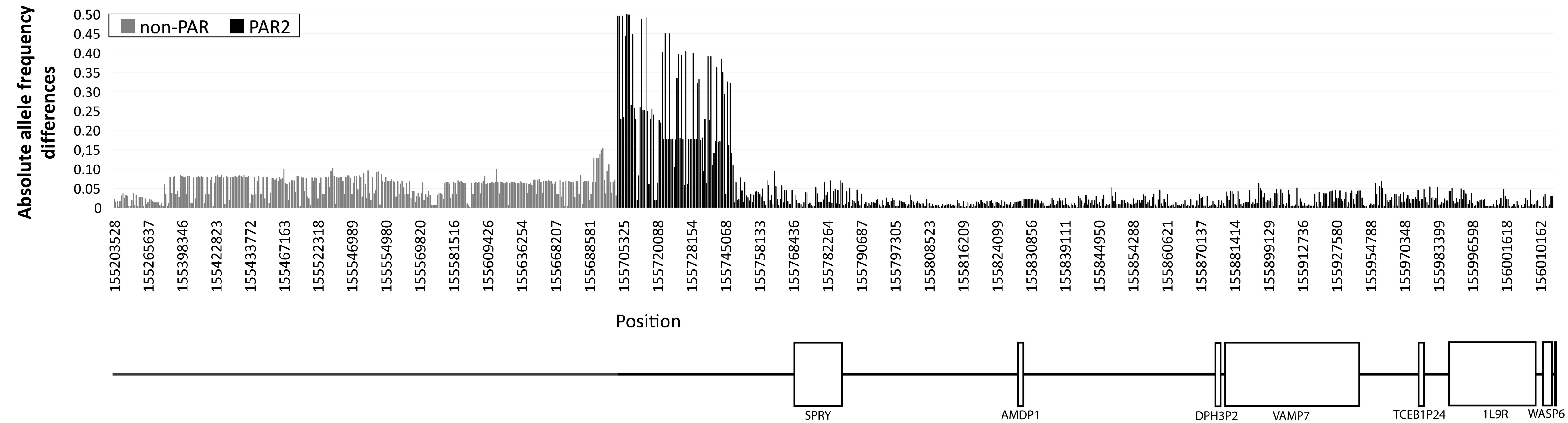

Supplement: S2 Fig — Absolute allele frequency differences between males and females for SNPs in PAR2 (black) and its flanking portion of the sex-specific region of the chromosome (grey) in the 1kGP African population data. (PDF) [file pgen.1009532.s007.pdf]

## PAR1

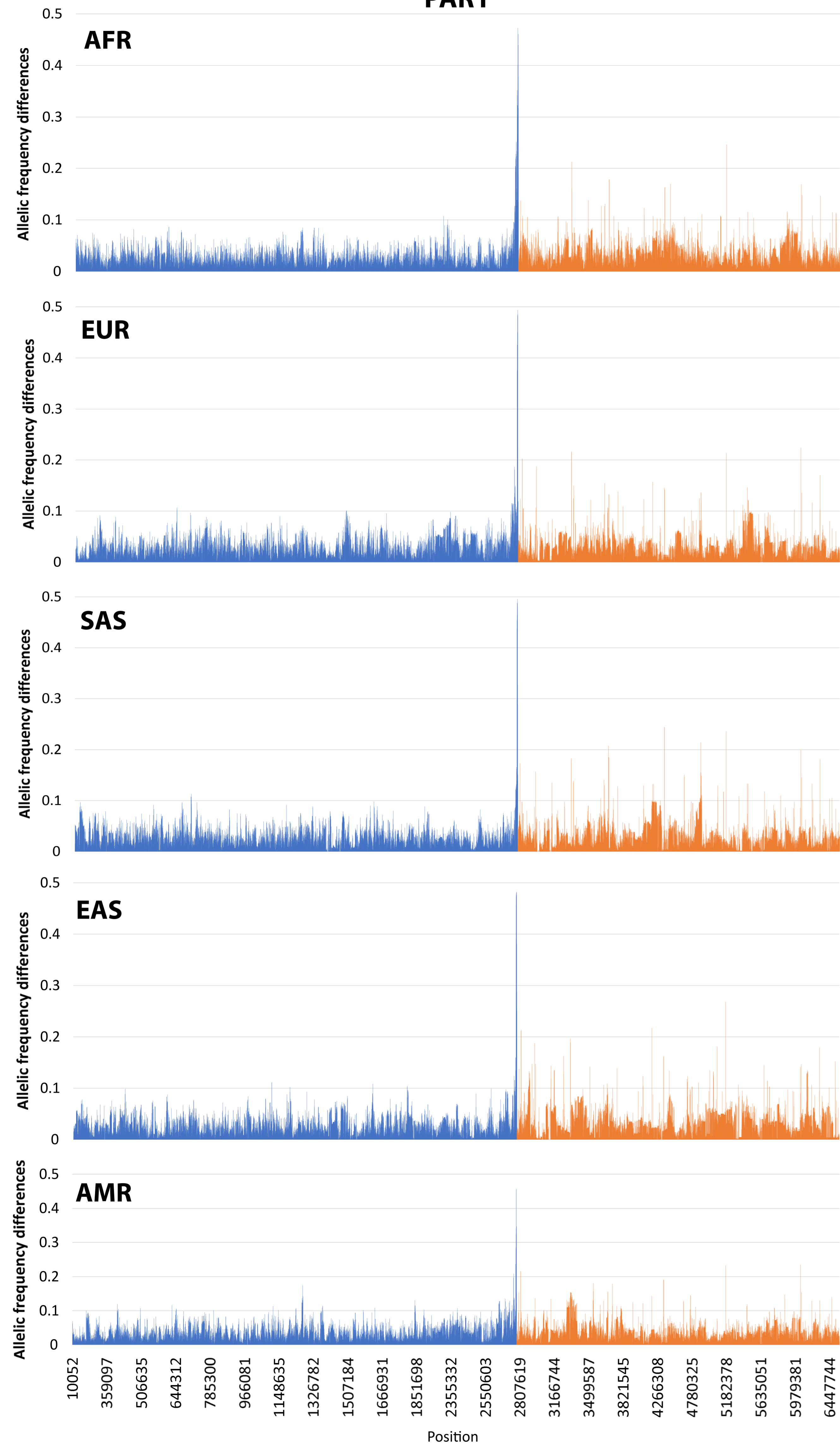

## PAR2

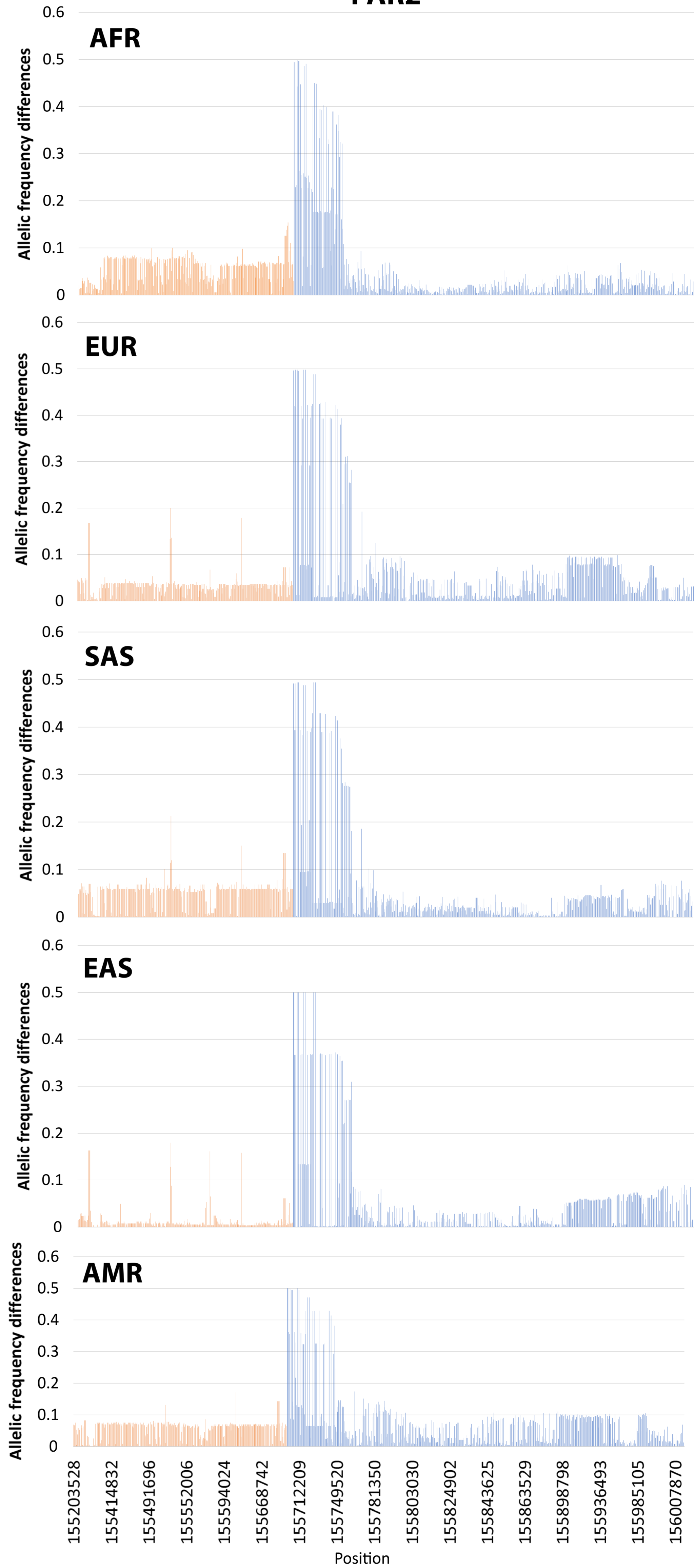

Supplement: S3 Fig — Absolute allele frequency differences between males and females for SNPs in the PARs (blue) and flanking portions (orange) in the five 1kGP super-populations. (PDF) [file pgen.1009532.s008.pdf]

(A)

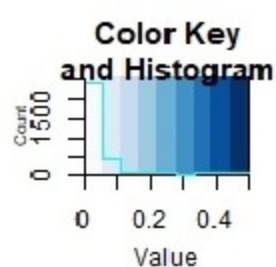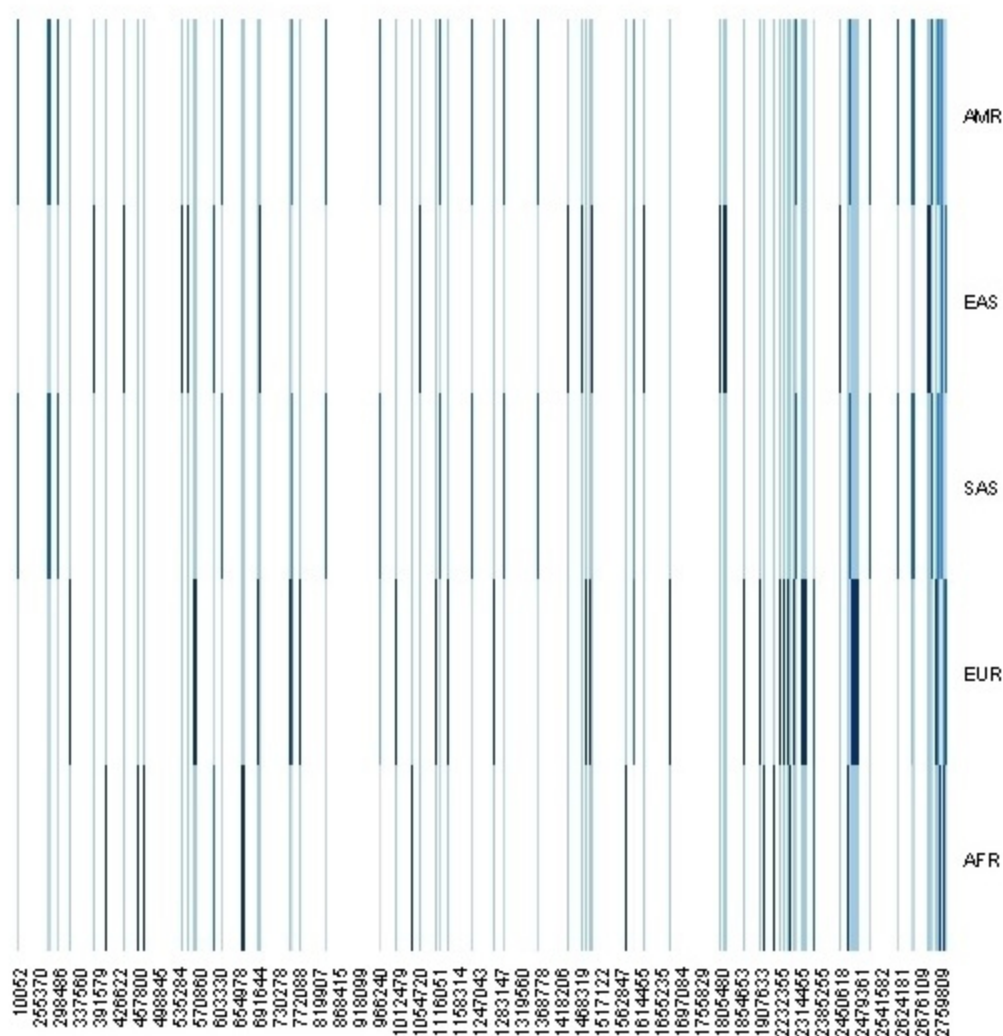

(B)

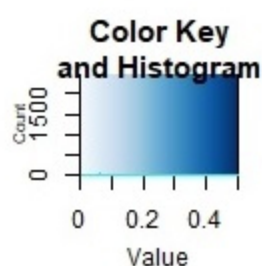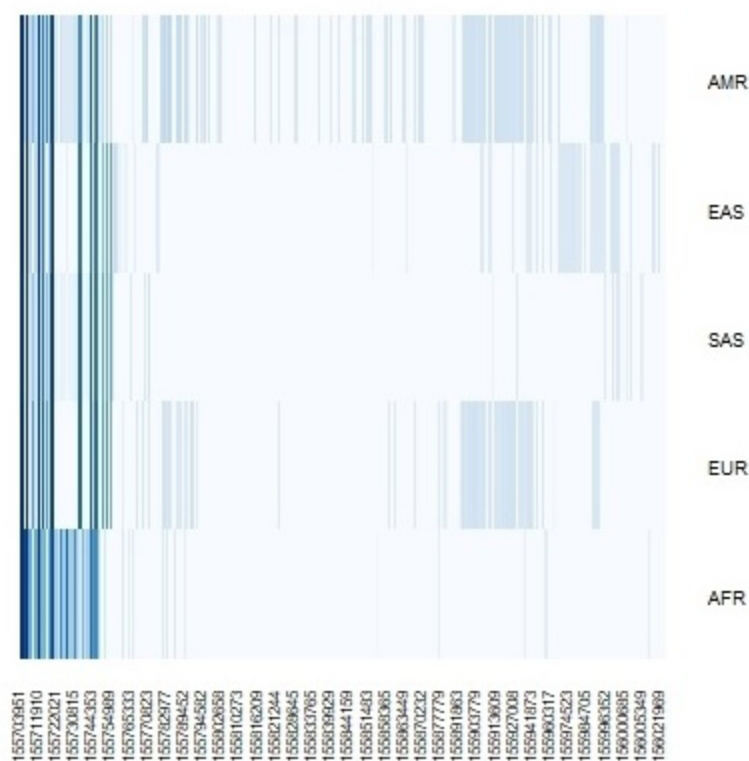

Supplement: S4 Fig — Heatmaps generated from allele frequency statistically significant differences between sexes for PAR1 (A) and PAR2 (B) in the different 1kGP super-populations. AMR: Admixed Americans; EAS: East Asians; SAS: South Asians; EUR: Europeans; AFR: Africans. The blue tones correspond to the range of p values of the Fisher test obtained for allele frequencies differences, according to the color key in the figure. (PDF) [file pgen.1009532.s009.pdf]

**A**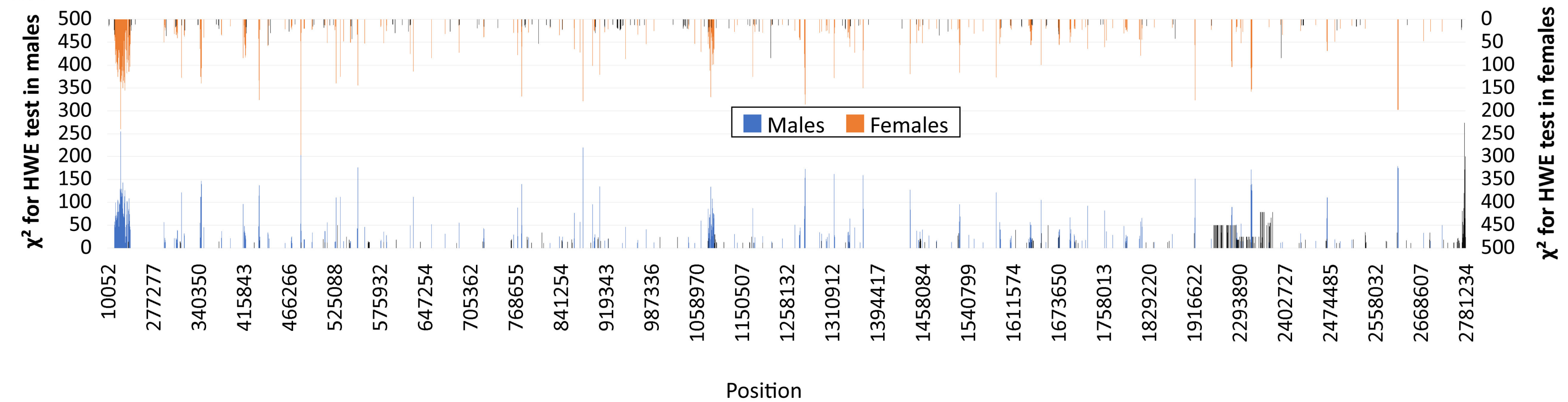**B**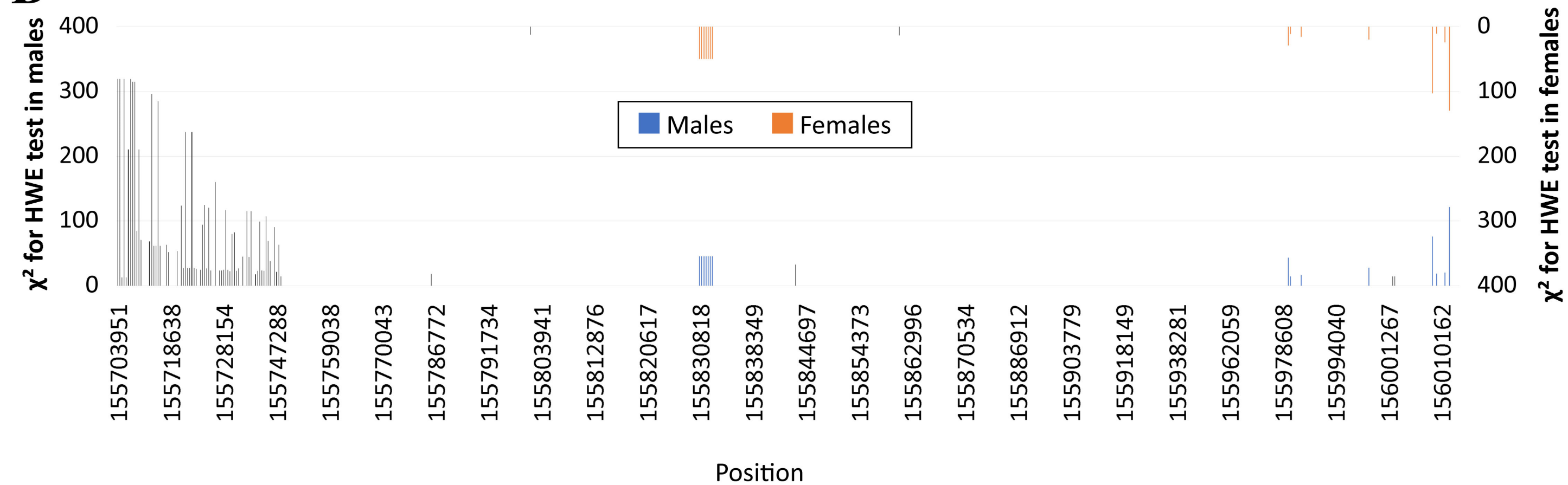

Supplement: S5 Fig — χ2 values of HWE tests for PAR1 (A) and PAR2 (B) associated to p < 0.05 in males (bottom in the plots) and females (top in the plots). Statistically significant departures in both sexes are represented in blue and orange bars for males and females, respectively. Statistically significant departures in one sex only are shown in black bars. (PDF) [file pgen.1009532.s010.pdf]

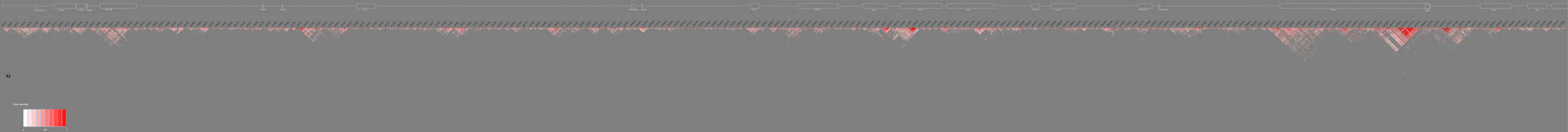

**X Chromosome**

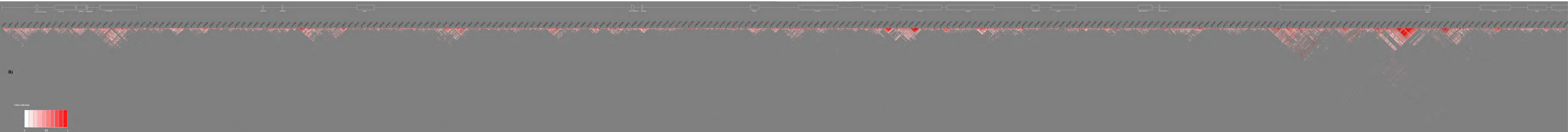

**Y Chromosome**

Supplement: S6 Fig — LD heatmaps based on r2 values for PAR1 at the X (A) and Y (B) chromosomes from the 1kGP African population. Each tone corresponds to different ranges of r2 values as represented in the color legend of the figure. (PDF) [file pgen.1009532.s011.pdf]

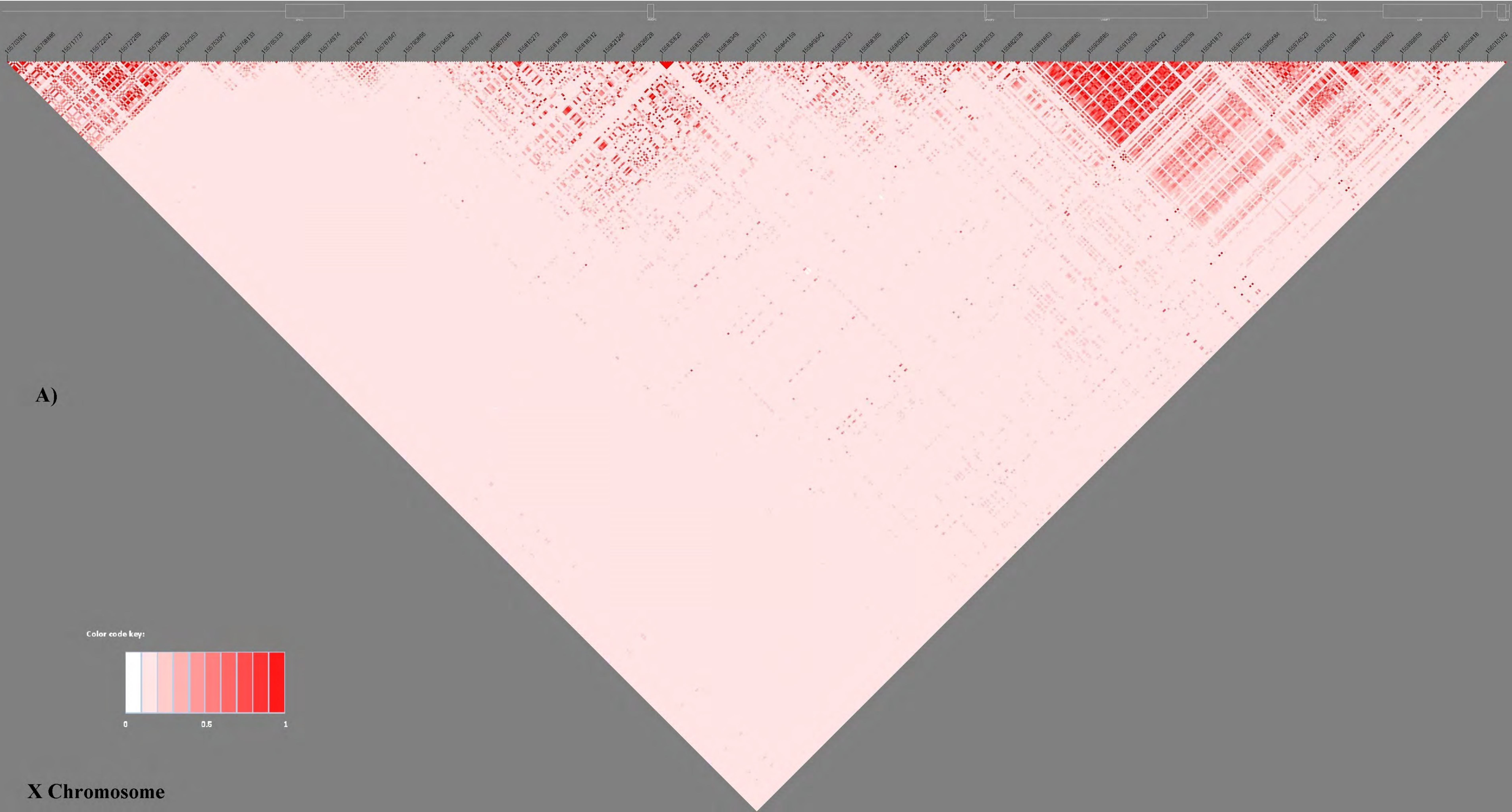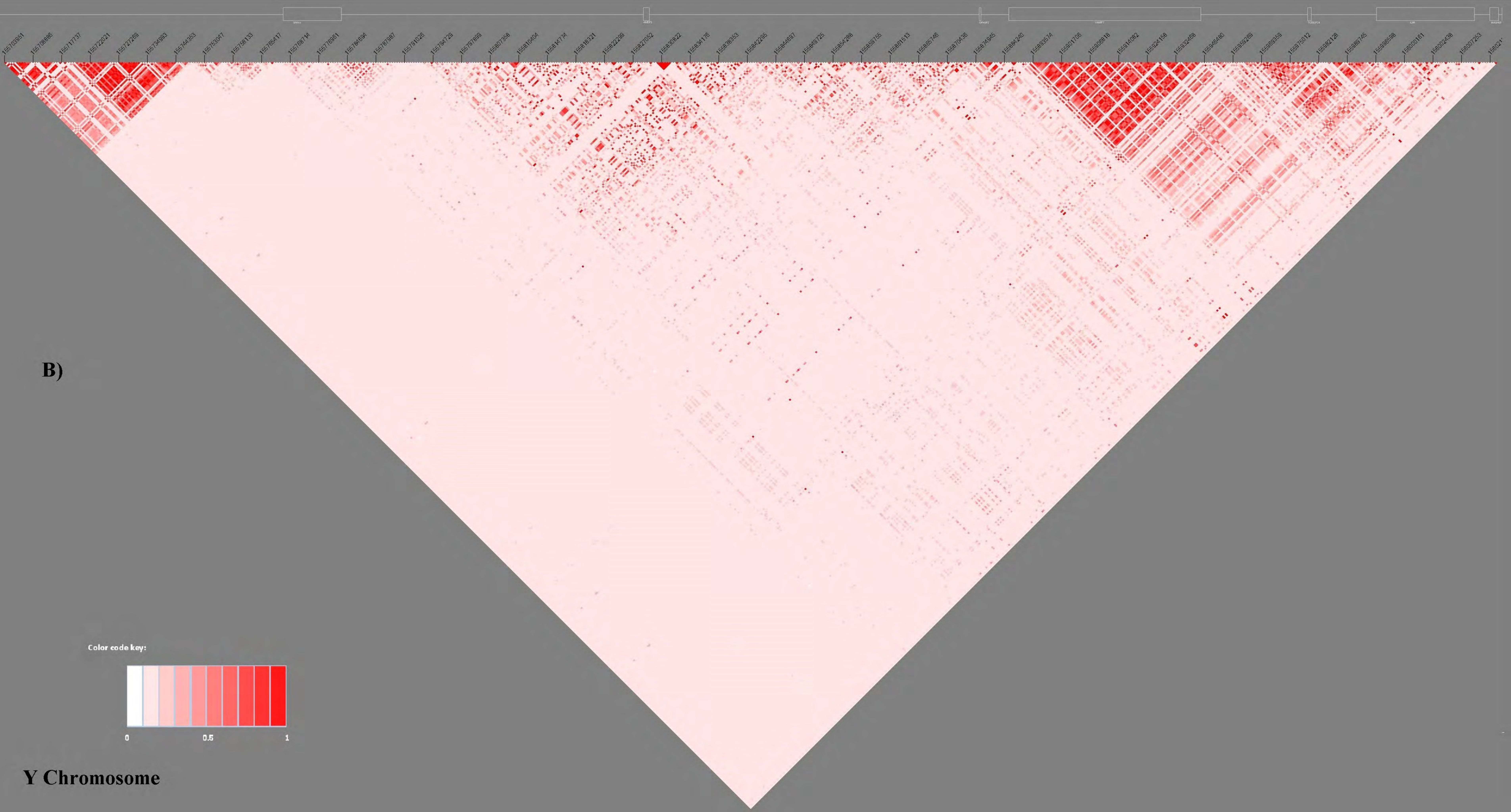

Supplement: S7 Fig — LD heatmaps based on r2 values for PAR2 at the X (A) and Y (B) chromosomes from the 1kGP African population. Each tone corresponds to different ranges of r2 values as represented in the color legend of the figure. (PDF) [file pgen.1009532.s012.pdf]

CD99

XG

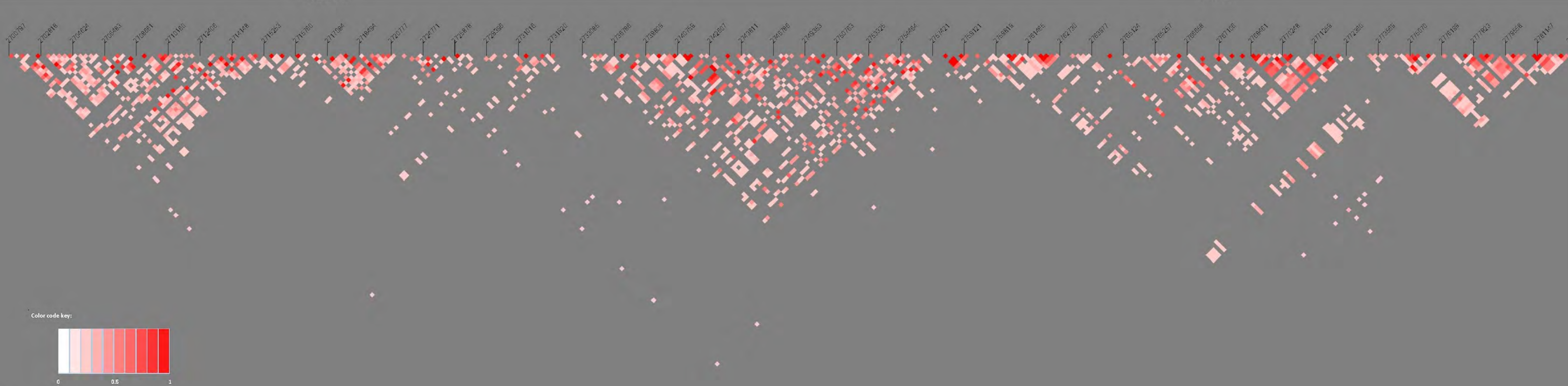

(A1) X chromosome: PAR1

SPRY

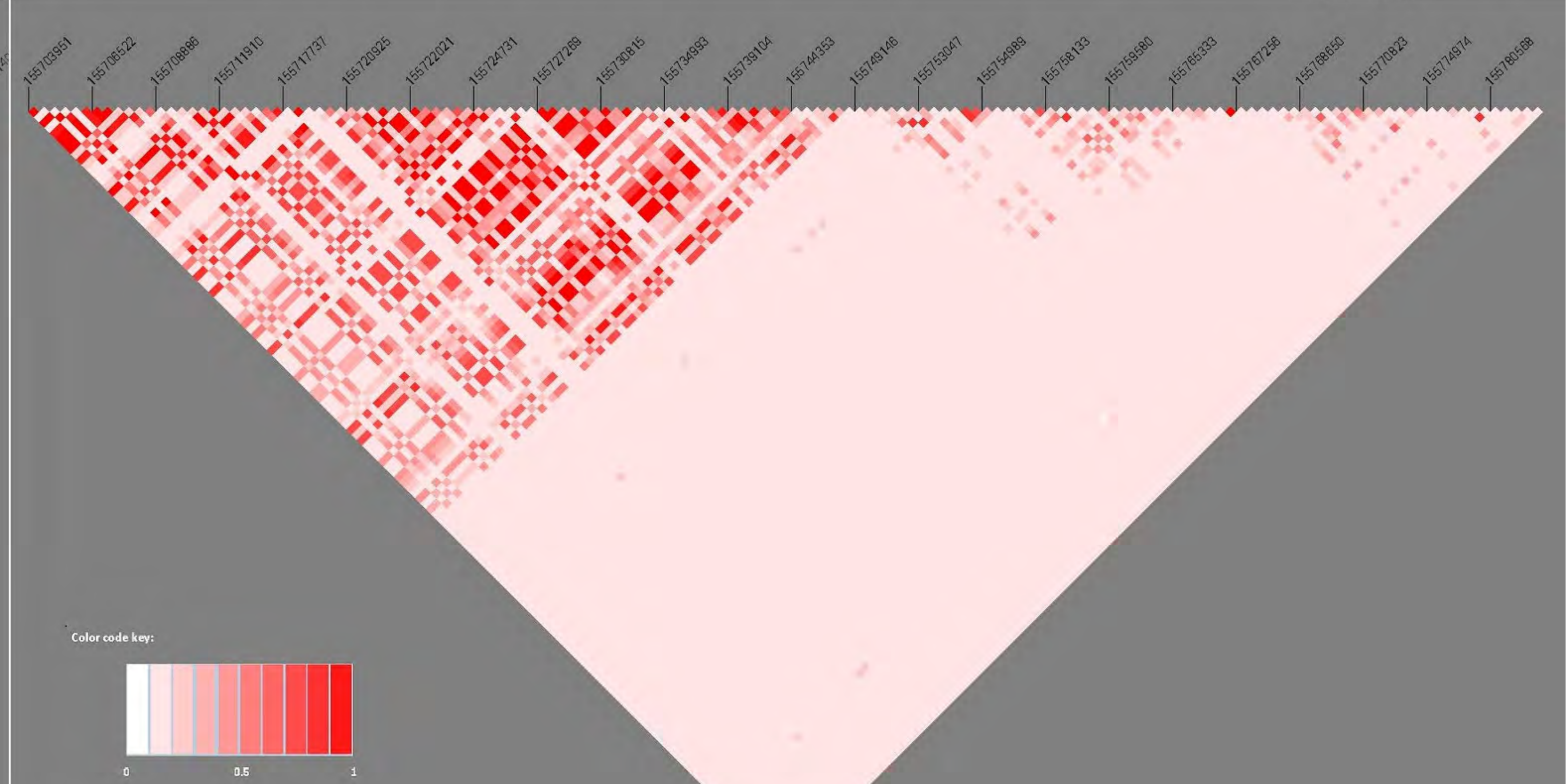

(A2) X chromosome: PAR2

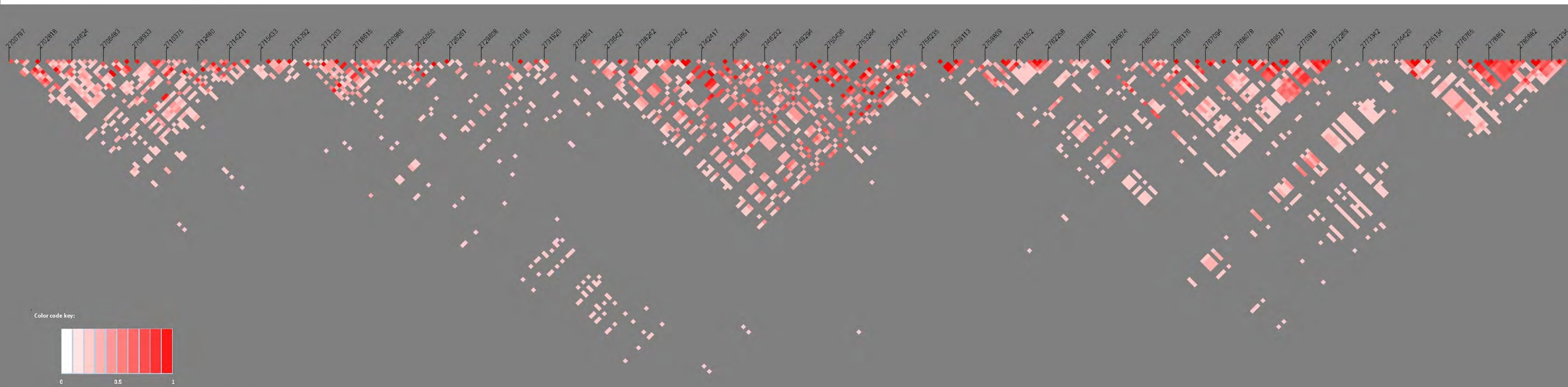

(B1) Y chromosome: PAR1

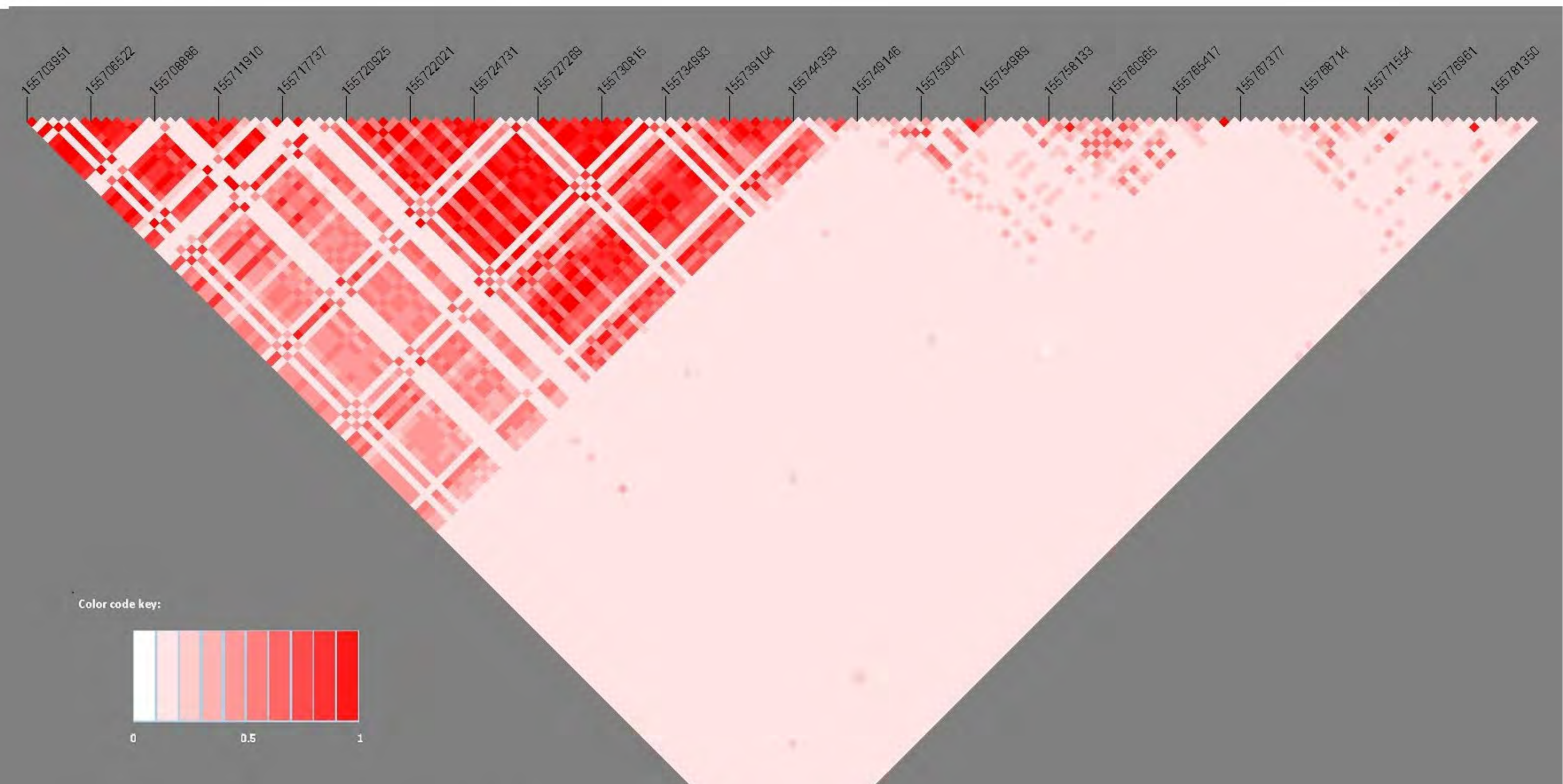

(B2) Y chromosome: PAR2

Supplement: S8 Fig — LD heatmaps based on r2 values at the X and Y chromosomes from the 1kGP African population. Each tone corresponds to different ranges of r2 values as represented in the color legend of the figure. (PDF) [file pgen.1009532.s013.pdf]

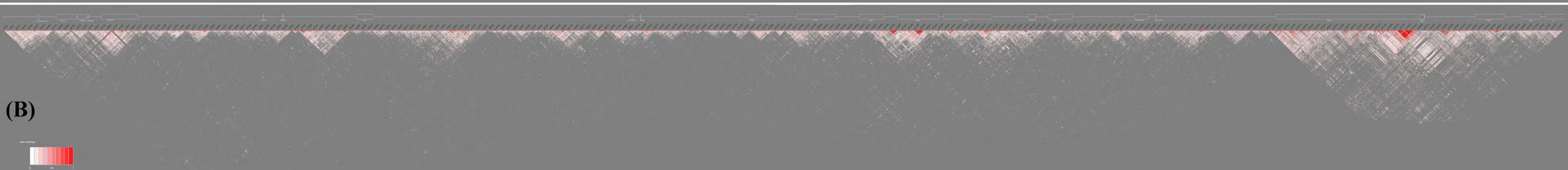

Supplement: S9 Fig — LD heatmaps based on r2 values in PAR1 at females (A) and males (B) from the 1kGP African population. Each tone corresponds to different ranges of r2 values as represented in the color legend of the figure. (PDF) [file pgen.1009532.s014.pdf]

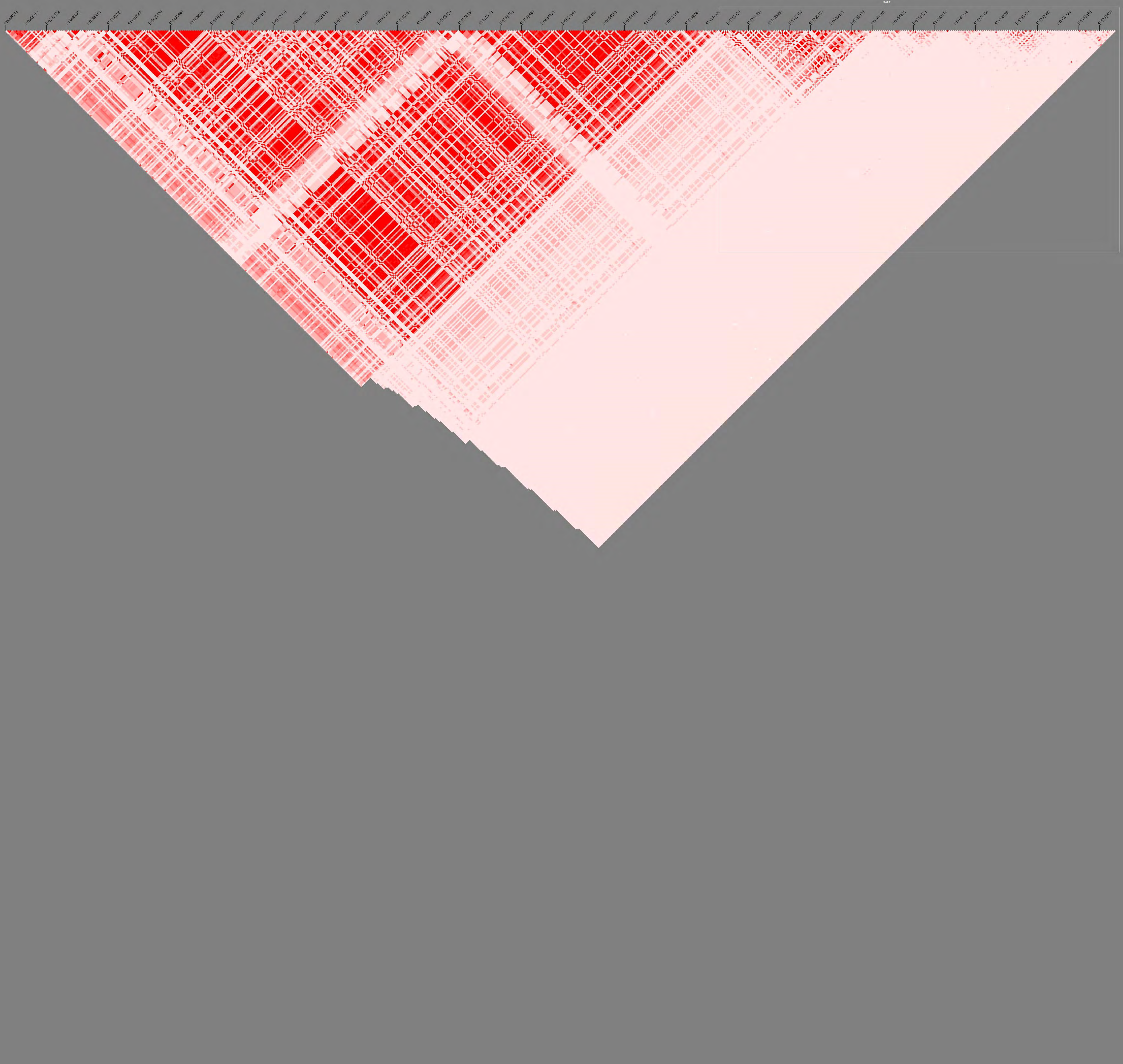

Supplement: S13 Fig — LD heatmap based on r2 values for PAR2 and the pseudoautosomal border region in females from the 1kGP African population. Each tone corresponds to different ranges of r2 values as represented in the color legend of the figure. (PDF) [file pgen.1009532.s018.pdf]

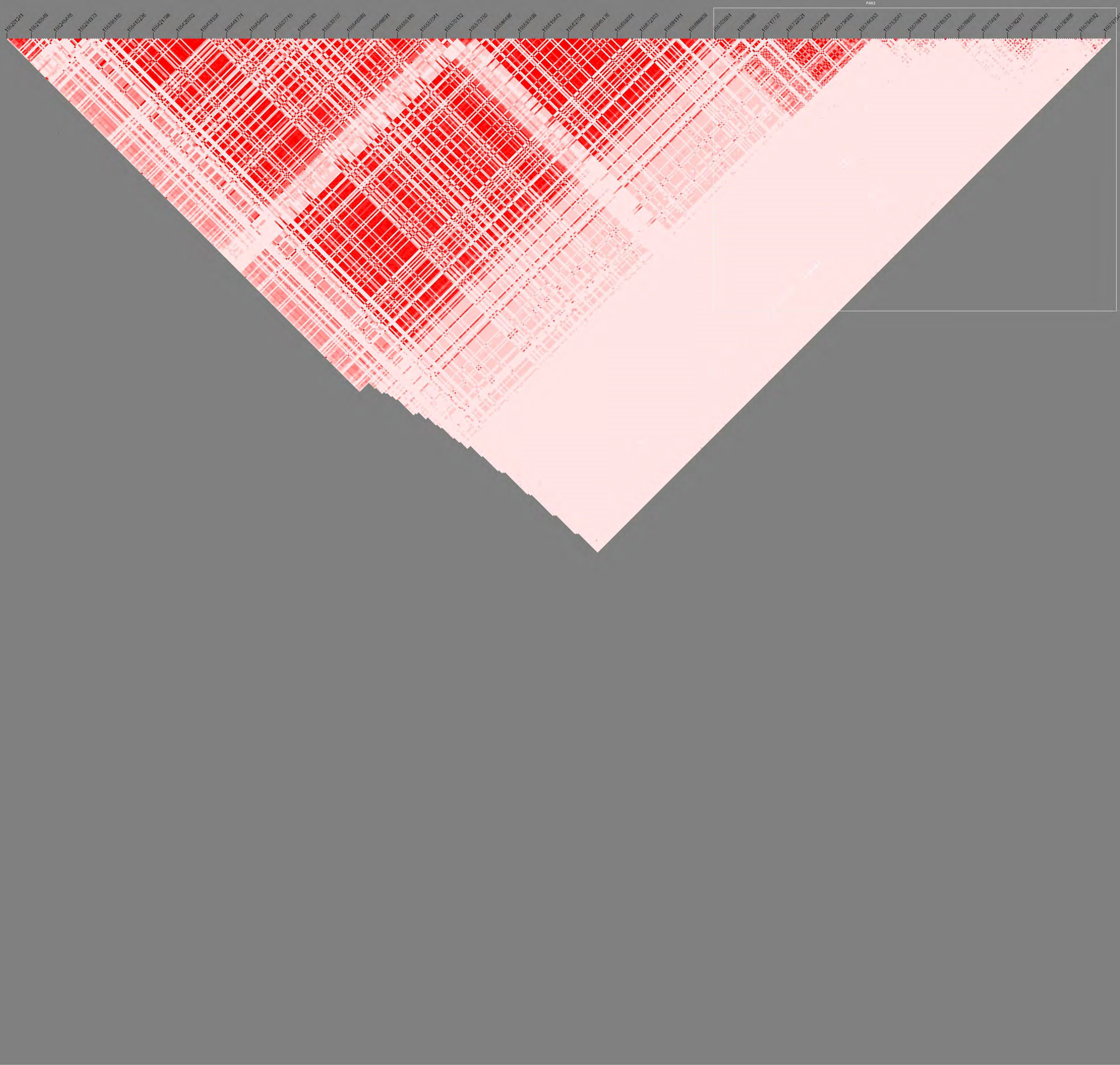

Supplement: S14 Fig — LD heatmap based on r2 values for PAR2 and the pseudoautosomal border region in males from the 1kGP African population. Each tone corresponds to different ranges of r2 values as represented in the color legend of the figure. (PDF) [file pgen.1009532.s019.pdf]
