# Supplementary material for: Evolutionary dynamics of the human pseudoautosomal regions
Source: PLoS Genet. 2021 Apr 19;17(4):e1009532. doi: 10.1371/journal.pgen.1009532 (PMC8084340; doi:10.1371/journal.pgen.1009532)

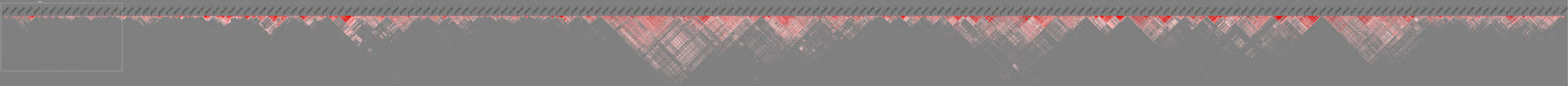

Supplement: S10 Fig — LD heatmap based on r2 values in PAR1 and the pseudoautosomal border region in females from the 1kGP African population. Each tone corresponds to different ranges of r2 values as represented in the color legend of the figure. (PDF) [file pgen.1009532.s015.pdf]
