# Supplementary material for: Evolutionary dynamics of the human pseudoautosomal regions
Source: PLoS Genet. 2021 Apr 19;17(4):e1009532. doi: 10.1371/journal.pgen.1009532 (PMC8084340; doi:10.1371/journal.pgen.1009532)
Supplement: S11 Fig — LD heatmap based on r2 values for PAR1 and the pseudoautosomal border region in males from the 1kGP African population. Each tone corresponds to different ranges of r2 values as represented in the color legend of the figure. (PDF) [file pgen.1009532.s016.pdf]

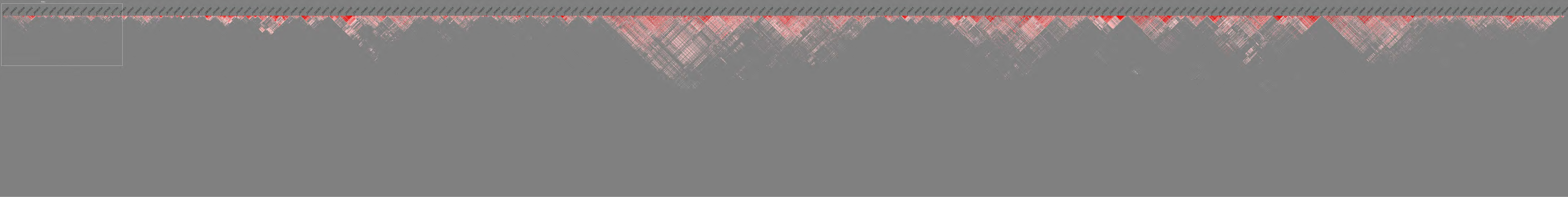
PAR1
2600980
2618503
2632658
2643832
2649606
2656935
2668791
2679134
2693458
2710375
2725444
2741963
2759094
2767106
2777789
2788903
2803605
2819241
2838372
2849145
2859273
2870666
2890568
2904560
2920983
2941551
2980085
