# Supplementary material for: Evolutionary dynamics of the human pseudoautosomal regions
Source: PLoS Genet. 2021 Apr 19;17(4):e1009532. doi: 10.1371/journal.pgen.1009532 (PMC8084340; doi:10.1371/journal.pgen.1009532)
Supplement: S12 Fig — LD heatmaps based on r2 values for PAR2 at female (A) and male (B) chromosomes from the 1kGP African population. Each tone corresponds to different ranges of r2 values as represented in the color legend of the figure. (PDF) [file pgen.1009532.s017.pdf]

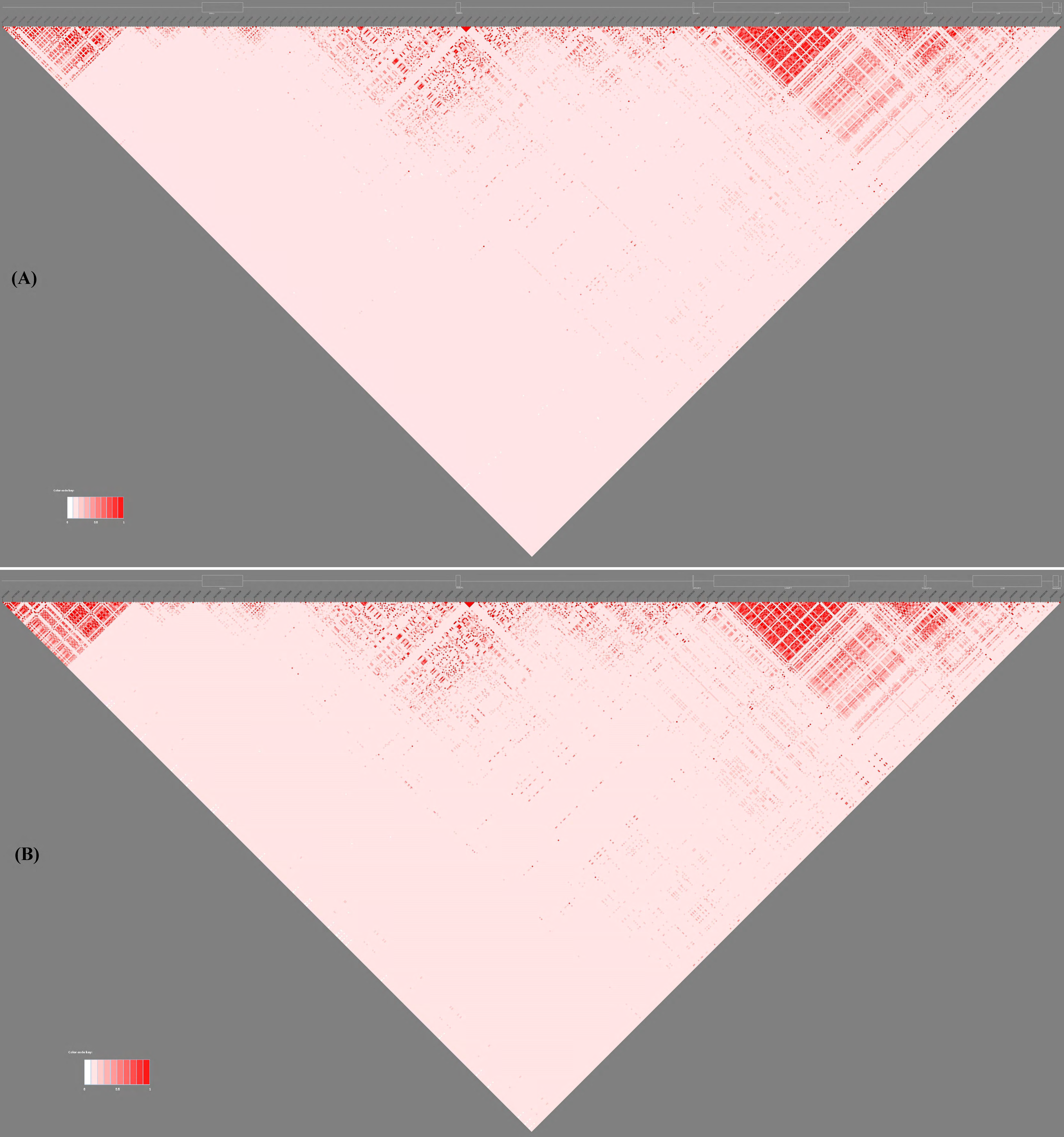
(A)
(B)
